# Large‐Area Atomically Flat Monocrystalline Gold Flakes: Recent Advances, Applications, and Future Potential

**DOI:** 10.1002/smll.202514856

**Published:** 2026-07-06

**Authors:** Amro O. Sweedan, Kefan Zhang, Muhammad Y. Bashouti, Thorsten Feichtner

**Affiliations:** ^1^ The Ilse‐Katz Institute for Nanoscale Science & Technology Ben‐Gurion University of the Negev Beersheba Israel; ^2^ Department of Solar Energy and Environmental Physics, Swiss Institute for Dryland Environmental and Energy Research, J. Blaustein Institutes for Desert Research Ben‐Gurion University of the Negev Midreshset Ben‐Gurion Israel; ^3^ Nano‐Optics and Biophotonics Group, Experimental Physics 5, Institute of Physics, Am Hubland University of Würzburg Würzburg Germany

**Keywords:** 2D crystalline gold, gold flakes, gold platelets, gold structuring, monocrystalline gold, nanotechnology, nano optics, nano photonics, plasmonics, sensing

## Abstract

High aspect ratio oblate polygonal gold crystals – such as triangular and hexagonal platelets – have attracted considerable interest due to their extraordinary physical, chemical, and mechanical properties. Commonly referred to as “gold flakes,” these structures exhibit atomically flat surfaces, µm^2^ areas with nanometric thickness, and a monocrystalline morphology. Since their first discovery by John Turkevich in 1951, considerable progress has been made in shape‐controlled synthesis and large‐scale production, unlocking steadily new opportunities for ever more advanced applications. This review explores large‐area gold flakes with lateral dimensions spanning from hundreds of nanometers to millimeters, with an emphasis on their unique properties. We provide a comprehensive overview of key developments, from early discoveries, synthesis approaches, and fabrication techniques to recent breakthroughs in the integration of gold flake as functional building blocks in photonics (e.g., for nanoantennas), sensing, nanoelectronics, biomedicine, and beyond. We conclude with a discussion of existing challenges, emerging applications, and possible future developments of this unique and versatile class of materials.

## Graphical abstract

This review surveys large‐area monocrystalline gold flakes, covering synthesis, growth mechanisms, and structural properties. It highlights their role as atomically flat, highly conductive platforms for precise nanostructuring, enabling applications such as plasmonics, sensing, and nanoelectronics. Current challenges are discussed together with future opportunities for integration into advanced nanoscale devices.

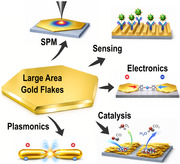

## 1 Introduction

**FIGURE 1:**
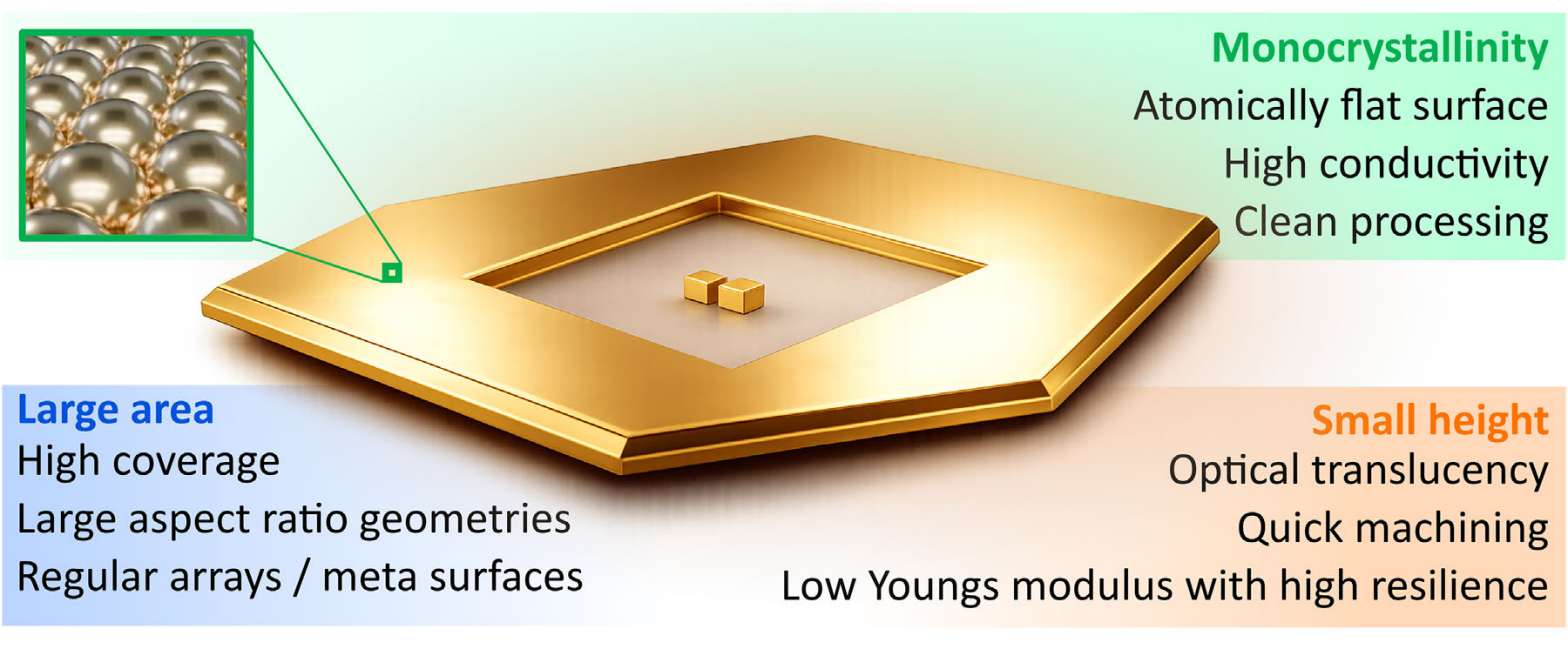
Gold flake properties overview. The three main properties of large aspect ratio oblate Au monocrystals and the main resulting benefits for their usage in applications.

Gold nanostructures, particularly wet‐chemically synthesized nanoparticles and large area nanofilms with a confined thickness, have garnered significant interest due to their unique physical, chemical, and optical properties [1, 2, 3, 4]. These materials have been widely utilized across diverse fields, including photonics [5, 6, 7, 8], electronics [9, 10, 11, 12], and diagnostics [13, 14, 15], among others [16, 17, 18, 19, 20]. Various possible morphologies of low‐dimensional gold have been reported, including spherical nanoparticles [18, 21], nanorods [22, 23], nanoflakes [24, 25], and other shapes [26, 27, 28, 29, 30]. Among these, a particularly notable class consists of high‐aspect‐ratio oblate nanostructures, typically triangular or hexagonal platelets, commonly and within this review called “gold flakes,” but also termed “gold platelets,” “gold prisms,” or “gold sheets” [31]. These particles are extraordinary due to their large area, atomically flat surfaces, monocrystalline nature, and therefore boundary‐free morphology, as well as their nanometer thickness. Figure 1 shows the consequential advantages that make gold flakes excellent candidates for many applications requiring, e.g., precise machinability, large conductivity, mechanical stability, or a combination of these [10, 32]. To this day, high‐yield synthesis with moderate to excellent control over the particle morphology has been well established [33, 34]. In particular, for flakes, a decent tuning of thickness and lateral dimensions has been demonstrated [8, 35].

This wet‐chemical bottom–up strategy for realizing atomically flat, thin metal films often can substitute conventional fabrication techniques, such as gold evaporation and subsequent lithography, milling, polishing, thinning, or template stripping, which inevitably produce polycrystalline films containing grain boundaries and structural defects that compromise material performance [6, 36, 37]. In contrast to traditional bulk mono‐crystal growth methods, which require subsequent mechanical thinning, cutting, or polishing to obtain nanoscale structures, chemically synthesized flakes are intrinsically several tens of nanometers thin and structurally defined. They therefore eliminate extensive post‐processing steps and provide a ready‐to‐use monocrystalline building block for nanoscale device architectures.

**FIGURE 2:**
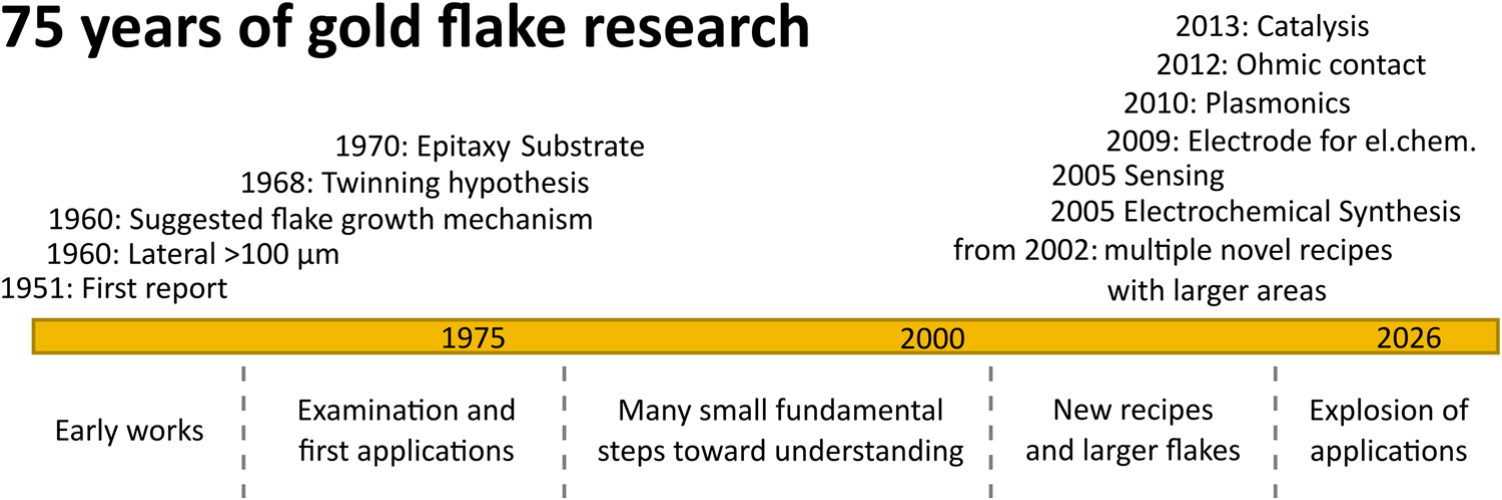
Gold flake research timeline. Evolution of the research and applications of large‐area monocrystalline gold flakes. The years are the first mention of an important concept or application in literature.

Hints of triangular and hexagonal gold particles date back as early as 1903 [38]. The first reliable observation of thin, monocrystalline gold flakes, however, was reported in 1951, when John Turkevich and co‐workers identified such structures as by‐products in complex reaction mixtures investigated by electron microscopy [39]. Surprisingly, similar flakes have also been reported to form naturally during drying events in saline groundwater [40]. An overview of the historical development of the subsequent research is sketched in Figure 2. In the following years after Turkevich's report, studies primarily focused on understanding the structural properties, crystalline structure, and growth mechanisms of these crystals, providing fundamental insights into facet‐controlled growth and confirming their predominantly monocrystalline nature, typically exhibiting only a small number of twin planes along the thin axis [41, 42, 43, 44, 45]. Over the following decades, research focused on understanding the formation mechanism of these structures, optimizing the synthetic protocols to reproducibly achieve large‐area and scalable production, and obtaining control on shape formation for unlocking the full potential of the flakes as universal building blocks for nano‐devices [6, 45, 46, 47, 48, 49]. To the best of our knowledge, the first high‐yield, shape‐controlled synthesis of noble metal flakes was reported in the early 2000s by Jin et al. [50], for silver. Shortly thereafter, independent research groups introduced various high‐yield methods for establishing controlled growth of gold flakes exceeding a diameter of a couple of hundred nanometers [51, 52, 53]. Today, gold flakes are synthesized using a range of different strategies, mostly variations of the classical wet‐chemical recipes [6, 10, 54], but also, e.g., physical methods [55, 56]. This newly achieved scalability triggered a large body of work demonstrating novel practical applications (see right side of Figure 2). Examples include the integration of top–down structuring techniques, stacking with other materials, and surface functionalization strategies, enabling applications in plasmonics, electronics, and sensing. These developments recently facilitated the realization of device‐level implementations based on gold flake architectures.

While extensive research on small‐area gold nanostructures exists [31, 57, 58, 59], studies on large‐area gold flakes ranging from micrometer to millimeter scale while retaining nanometric thickness confinement remain relatively limited until today. However, their increased utilization, whether employed as‐synthesized in a classical bottom–up fashion or further structured using top–down techniques such as focused ion beam (FIB) milling, will benefit from a comprehensive collection of the existing knowledge as a starting point for the next decades of gold flake‐based research. This review summarizes the structural and physical properties of large‐area gold flakes, the various synthetic methodologies, and their applications in photonics (especially plasmonics, where the gold flakes were adopted first as a basis for further structuring [6]), as well as in (bio)sensing, nanoelectronics, among others, and their potential in next‐generation technologies. Selected applications are highlighted that represent the progress in the field, whereas an extensive overview of available studies is compiled in structured tables to provide a complete and accessible account of the field.

In this review, we primarily focus on the intrinsic structural, optical, and electronic properties of the gold flakes themselves, while application‐specific mechanisms are discussed in a concise and summarizing manner. The physical and chemical mechanisms underlying the applications of gold flakes are numerous and highly diverse. A comprehensive treatment of all possible mechanisms benefiting from this material is beyond the scope of the present work. Interested readers are therefore referred to the extensive bibliography for detailed discussions of the underlying physical and chemical processes.

## 2 Gold Flake Structural Properties and Growth Mechanism

**FIGURE 3:**
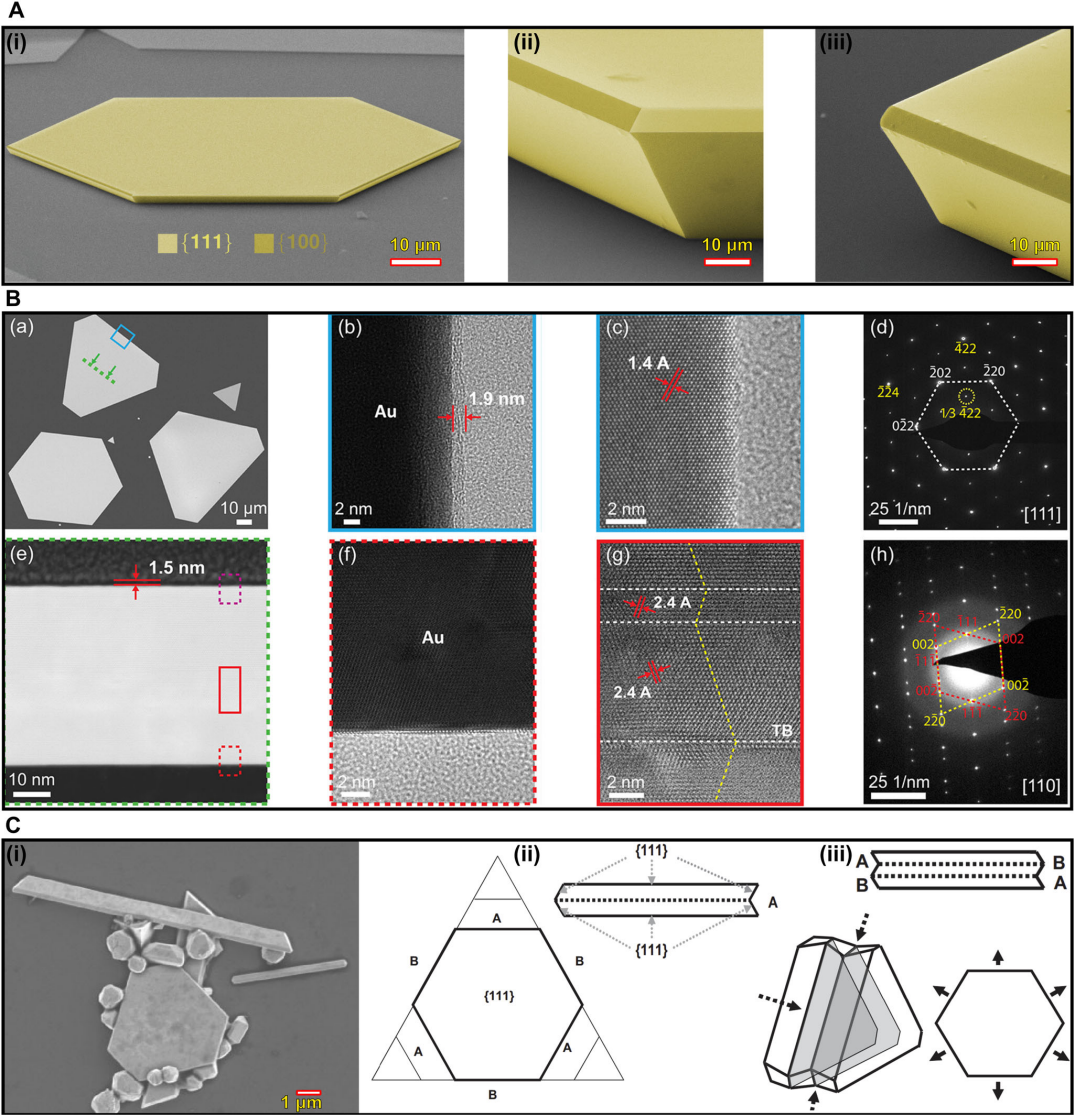
Structural properties of gold flakes. (A_i_) SEM image of an Au monocrystalline flake tilted at a 75° angle. (A_ii_‐A_iii_) Close‐up high‐resolution SEM images of two corners of the flake, also tilted at 75°. Artificial coloration highlights different crystallographic planes on the facets: light yellow for {111} and dark yellow for {100}. Panels (A) adapted from Boroviks et al. [84]. Copyright 2018, Optical Society of America. (B) Microstructural and crystallographic properties of gold flakes. (B_a_) Top‐view SEM image of gold flakes. (B_b_) Planar‐view bright‐field TEM image of the edge of a solution‐grown gold flake, showing a 1.9 nm thick organic layer on the side facet. (B_c_), (B_d_) Atomic‐resolved HRTEM image and selected area electron diffraction pattern (SAED) of the same region. (B_e_) Cross‐sectional STEM image of a flake after FIB cross‐sectioning. (B_f_) Atomic‐resolved HRTEM image of the flake–glass substrate interface region. (B_g_) Higher magnification HRTEM image and (B_h_) SAED pattern of the twinned region of the flake, with 2.4 Å spacing between the {111} planes of fcc‐Au, twin boundaries, and mirrored diffraction spots. Panel B Adapted from Kiani et al. [66]. Copyright 2022, The Authors. (C_i_) Diversity of shapes synthesized in a homogeneous reaction environment, including rods, tapes, flakes, tetrahedra, and isotropic particles. (C_ii_) Sketch of a single twin plane Au flake, where alternating sides display A‐type and B‐type faces. The reentrant grooves on the A‐type faces promote rapid growth, which is halted when the face grows out, leaving behind a triangular prism with slower‐growing B‐type faces. (C_iii_) Sketch with two parallel twin planes. All six sides display A‐type faces (denoted by dashed arrows) with reentrant grooves. This enables each A‐type face to regenerate those adjacent to it, facilitating rapid growth in two dimensions (solid arrows). Panels (C) reprinted with permission from Lofton et al. [68]. Copyright 2005, WILEY‐VCH Verlag GmbH & Co. KGaA, Weinheim.

Gold flakes, along with less prevalent geometrical variations such as nanosheets and nanodisks [6, 10, 54], can generally be described as oblate nanocrystals, often exhibiting rotational symmetry about their short axis, most commonly approximating C3 (triangular) or C6 (hexagonal) symmetry depending on their morphology (see, e.g., Figure 3A_i_,B_a_). Geometrically, these crystals are confined by two parallel polygonal surfaces, with lateral dimensions ranging between a few nanometers and millimeters [37, 60]. Owing to their dimensions, which laterally often exceed several hundred nanometers and vertically rarely go below 20 nm, the bare flakes typically do not exhibit phenomena associated with nanoscale confinement and can be described entirely using classical models [1, 61, 62, 63], (Comments on quantum properties are collected in the next section). The crystal structure of solution‐prepared gold flakes is typically monocrystalline, exhibiting a face‐centered cubic (fcc) lattice [31, 52, 64, 65, 66], with a boundary‐free configuration along their lateral plane [36, 67]. The upper/lower facets are composed of nearly atomically {111} crystal faces [64, 65, 66, 68]. Edges commonly expose {100} or {110} planes, high‐index facets, or even {111}, depending on the flake's thickness and specific growth conditions [2, 68, 69], (see Figure 3A). High‐resolution transmission electron microscopy (HRTEM) and convergent beam electron diffraction (CBED) analysis both show that gold flakes contain at least one twin plane parallel to their {111} surfaces (Figure 3B). This twin boundary is a result of an initial atomic‐scale stacking fault, where the fcc lattice inversion creates a symmetrical plane of reflection across the flake plane [65, 66, 68, 70], (Figure 3C_ii_,C_iii_). This is one origin of the enhanced in‐plane growth of the flakes during synthesis (a simulation respecting this principle can be found here [71]).

According to LaMer's nucleation model, after the reduction of gold ions, the resulting gold atoms reach a supersaturation threshold before spontaneously forming small clusters, which then evolve into stable nuclei [72]. Surface energy considerations result in favor of {111} facets, leading to the formation of symmetrical seeds, minimizing free energy [73]. Anisotropic shapes will be obtained only when this rule is broken, either by lowering the surface energy of particular facets (thermodynamic control) or by reducing the relative growth rate of competing facets (kinetic control). This can be achieved by employing capping agents that selectively regulate growth rates across different facets while gold ions are being reduced in a kinetically controlled pathway [34, 49, 74]. Experimentally, an efficient capping agent changes the order of free energies for different crystallographic facets of gold nanocrystals through selective chemisorption [67, 74]. Capping agents include surfactants, small molecules, atomic adsorbates, and biomolecules with molecular recognition capabilities [34, 75]. Alternatively, a template can impose external geometric constraints, accelerating seed formation and guiding crystal growth along specific directions [76], leading to anisotropic morphologies even in the absence of capping agents. Specifically, rapid nucleation induced by the template's constraints can lead to the formation of twinned seeds, where internal structural defects naturally promote anisotropic growth without the need for additional shape‐directing agents [66, 76].

An example of a capping agent is polyvinylpyrrolidone (PVP), a strongly binding surfactant preferentially onto Au {111} facets, suppressing a crystal's vertical growth while promoting lateral expansion [77]. Similarly, halide ions (I^−^, Br^−^, Cl^−^) exhibit a high affinity for {111} surfaces, directing nanoplate growth while also functioning as etchants that eliminate undesired nucleation sites [66, 78, 79]. The final morphology of gold flakes is therefore influenced by reaction parameters such as precursor concentration, reduction kinetics, and temperature [31]. A slow reduction rate after the nucleation phase favors the controlled deposition of gold atoms onto pre‐existing seeds, promoting the formation of large‐area flakes [34, 80]. Additionally, lateral diffusion rates on {111} facets exceed vertical stacking rates under the influence of surfactants, further enhancing the formation of 2D nanostructures [68]. The self‐assembly of small nanoflakes into larger crystalline domains has also been observed, driven by high surface energy along lateral facets [81, 82].

The formation of multiple shapes (see Figure 3C_i_), such as triangular or hexagon flakes in a single homogeneous reaction, as well as their occurrence across different synthesis methods was explained by Lofton et al. [68], and attributed to the formation of different amounts of twin plane defects during initial nucleation, which yield both concave (A‐type) and convex (B‐type) surface features along the nanoplate edges (Figure 3C_ii_,C_iii_). The interplay between the faceting tendency of the two types dictates the final morphology. With this understanding, researchers have successfully synthesized large‐area monocrystalline gold flakes with geometric control for further applications [8, 33, 34, 35]. In addition to the coexistence of multiple shapes within a single reaction, Großmann et al. [83], reported stepped thickness variations occurring within individual flakes. The underlying growth mechanism remains unresolved until now, with diffusion limitation currently proposed as the working hypothesis.

## 3 Notes on Quantum Properties

Gold flakes are becoming a playground for fundamental quantum mechanics research. This is due to their atomically smooth surface, their possibly sub‐10 nm thickness when mesoscopic properties begin to show up, and recent fabrication schemes to realize single to sub‐nanometer geometrical features, e.g., by helium ion milling or stacking with 2D materials. In the following, we quickly introduce the resulting effects known so far, so that the reader can delve deeper if he wants to get into this emerging topic.

The first class of quantum effects is due to the electron energy levels becoming discrete for flake thicknesses below 2.5 nm [85]. The system then starts to behave like a quantum well [86]. There are claims that quantum effects start already at larger thicknesses as measured in nonlinear photoluminescence [83], but there are also purely classical models available working with lowered heat conductivity instead [87].

The second class of quantum effects concerns (atomically flat) metallic surfaces. When the experiment is very well defined, it is possible to study the thin transition region from a metal half space filled with conduction electrons to a half space of dielectric/air/vacuum which spans only a few Angstroms. The electron density at the surface is not discontinuous—as often and often reasonably approximated—but has a smooth (continuously differentiable) wave function leaking out of the metal, which is coined “electron spill out”. Theories capturing this effect are first‐principle methods like time‐dependent density functional theory (TD‐DFT), which is computationally expensive and only work until now for systems as small as a few thousand electrons [88], or thin metal slabs in periodic boundary conditions [89]. Alternatives for larger systems are based on hydrodynamic/jellium models [90].

The most successful way to describe surface quantum effects is using surface response functions (Feibelman d‐parameters) [91, 92, 93]. Here, surface currents and surface dipoles are approximated by an infinitely thin layer using integration perpendicular to the surface. The resulting d‐parameters can then be incorporated into classical Maxwell's equations. There is a lot of research happening at this very moment to make this tool work as a connection between theory and experiment. On the theoretical side, effects like non‐locality [94], and Landau damping [95], are evaluated in terms of surface currents and charges. Also not clear is the influence of topological surface states on crystalline gold surfaces [96] on the surface response.

Finally, the last class of quantum effects to be considered is happening in (ultra‐)small gaps. Non‐locality becomes a big influence, recently evaluated in theory and experiment for large area gaps [97]. Also, elastic and inelastic tunneling emerges, and with it, smaller optical near field enhancements, the novel charge transfer mode reaching over the gap for elastic tunneling, but also a new radiative and non‐radiative loss channel via inelastic tunneling. Several review articles describe the underlying physics [98, 99, 100]. A recent review of quantum mechanical descriptions of metallic nanoparticles can be found in Ding et al. [101].

## 4 Synthesis Routes and Manipulation of Large‐Area Gold Flakes

In the last two decades, various synthesis approaches have been developed to realize gold flakes. Despite their diversity, all methods follow a common principle: promoting lateral growth while restricting vertical expansion to realize a thickness confinement [69, 74, 102, 103].

Gold flakes synthesis typically relies on the reduction of gold precursors, where gold ions (Au^3^^+^) are converted into their neutral metallic state [10, 54]. This process can be achieved through classical wet‐chemical routes [6, 10, 36, 54], physical methods [55, 56], bioinspired techniques [52, 104], and hybrid approaches that combine multiple methodologies [105, 106].

Synthesizing large‐area gold flakes requires a precise balance between thermodynamic driving force and kinetic control. Under purely thermodynamic equilibrium, gold tends to adopt compact morphologies that minimize total surface energy, typically resulting in quasi‐spherical shapes of fcc crystals [74]. To instead achieve two‐dimensional (2D) morphologies, a clear separation of nucleation and subsequent growth conditions is required [26]. Control over the synthesis process begins with the reduction of gold precursor ions (e.g., AuCl_4_^−^) to zerovalent gold atoms through high initial concentration and/or temperature, which promotes rapid nucleation and seed formation [71]. This stage is primarily governed by kinetic factors, while thermodynamic driving forces, such as surface energy minimization, become more relevant during subsequent growth and shape evolution [74, 107]. Under appropriate conditions, planar defects such as twin boundaries are introduced within these seeds. These defects, combined with the intrinsic surface energy anisotropy of gold (with low‐energy {111} facets), suppress growth in certain directions and promote lateral expansion. As a result, plate‐like or flake morphologies develop. Achieving such 2D structures, therefore, relies on kinetically controlled growth pathways that deviate from the thermodynamically favored isotropic morphology [74, 108].

**FIGURE 4:**
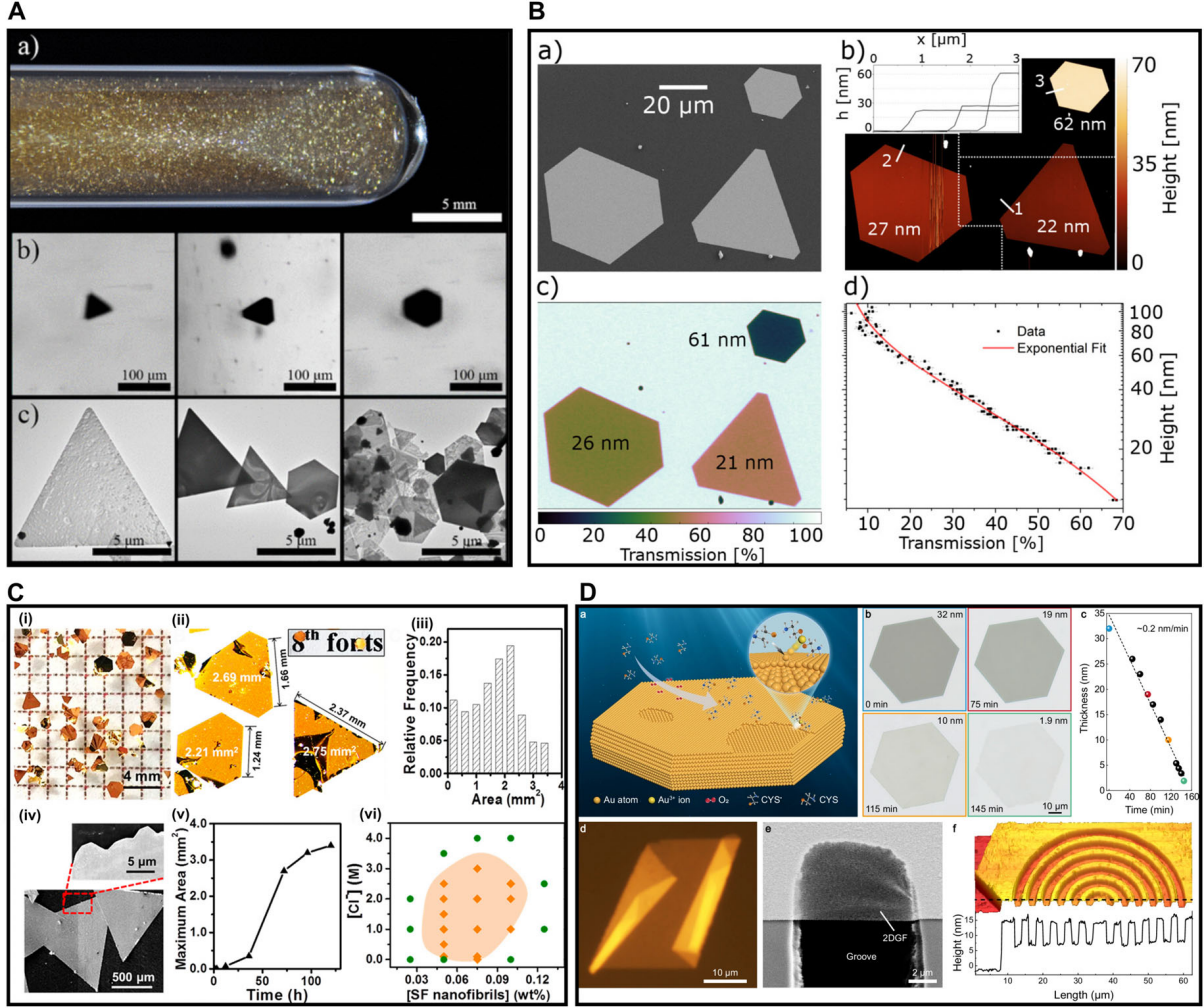
Gold flake synthesis approaches. (A) Matrix‐based (template‐based) chemical synthesis of gold flakes. (A_a_) Optical photograph of the gold flakes inside the matrix; the appearance of bulk gold optical property is visible as the flake size grows from left to right. (A_b_) Optical images of synthesized flakes. (A_c_) TEM images of extracted flakes. Adapted from Farkas et al. [114]. Copyright 2021, The Authors. (B) On the substrate, large‐area gold flakes were synthesized. (B_a_)‐(B_c_) SEM, AFM, and optical transmission images of the individual gold flakes. (B_d_) Optical transmission as a function of flake thickness. Reprinted (adapted) with permission from Krauss et al. [76]. Copyright 2018, American Chemical Society. (C) Millimeter‐scale gold flake synthesized biochemically using nanofibrils. (C_i_), (C_ii_) Optical images of millimeter‐sized gold flakes alongside a size comparison with an 8‐point Roman font. (C_iii_) Histogram showing planar area distribution. (C_iv_) SEM images of gold flakes during synthesis, the zoom showing a growth region. (C_v_) flake area against growth time, (C_vi_) Concentration window of SF nanofibrils and Cl^−^ to synthesize 2D Au crystals; Au crystal surface area > 1.0 mm^2^ = orange; area < 1.0 mm^2^ = green. Reprinted (adapted) with permission from Lv et al. [60]. Copyright 2018, American Chemical Society. (D) Generation of ultrathin (single‐digit nanometer) gold flakes via chemical etching. (D_a_) Schematic of the etching process. (D_b_) Optical transmission micrographs at different etching times. (D_c_) Thickness of the gold flake as a function of etching time. (D_d_) Optical reflection micrograph showing a folded gold flake. (D_e_) SEM image of a gold flake suspended over a groove. (D_f_) AFM image of a locally etched gold flake patterned into concentric rings. Adapted from Pan et al. [37]. Copyright 2024, The Author(s).

**FIGURE 5:**
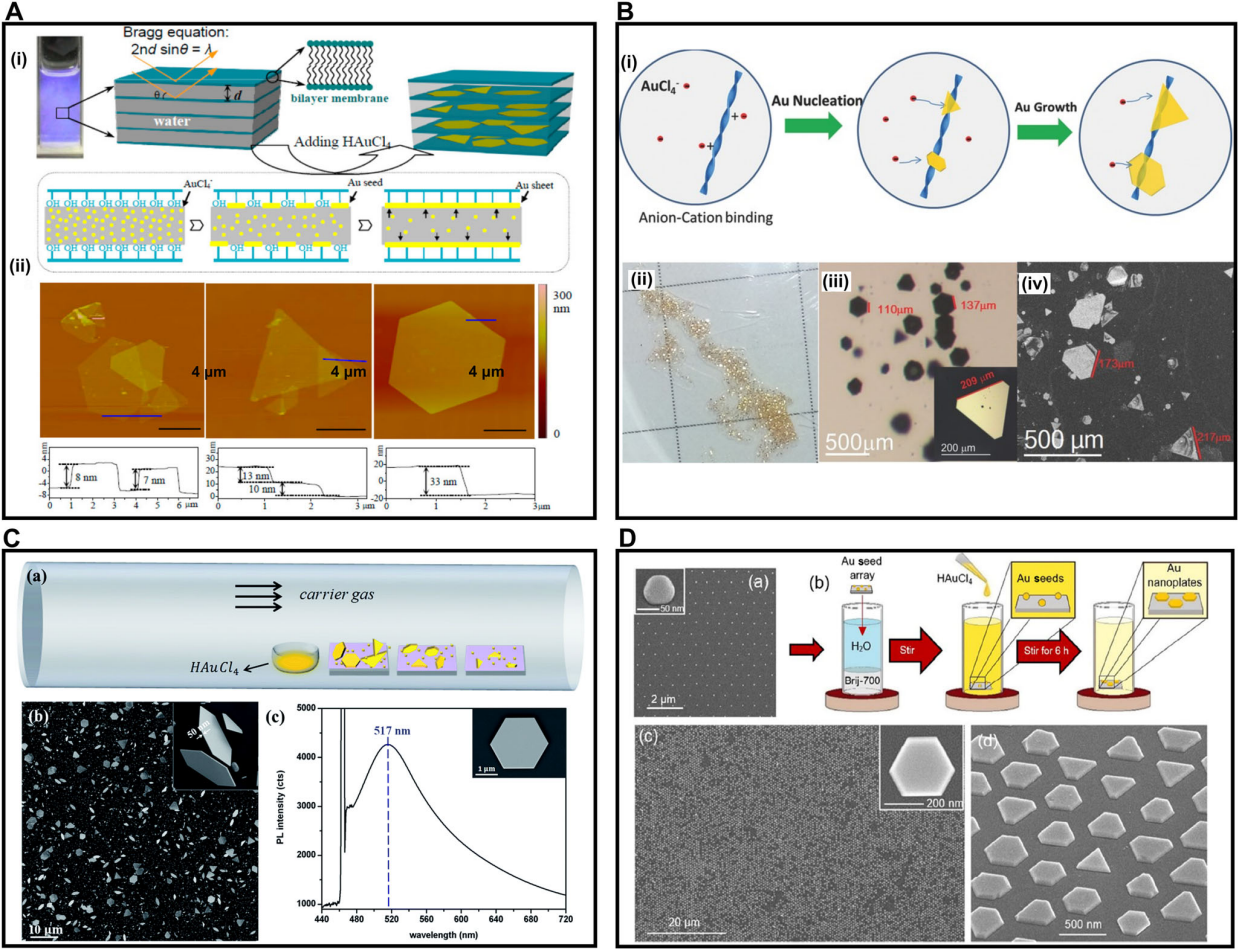
Large‐area gold flake synthesis: advanced strategies. (A) Thickness‐controlled directed synthesis of gold flakes. (A_i_) Schematic procedure describing the water layers sandwiched directed synthesis of gold flake by lamellar bilayer membranes of a self‐assembled nonionic surfactant. (A_ii_) AFM scans of gold flakes obtained at different precursor concentrations and corresponding height profile curves. Reprinted (adapted) with permission from Qin et al. [35]. Copyright 2013, American Chemical Society. (B) Bioinspired synthesis of macroscopic monocrystalline gold flakes using amyloid fibrils as templates. (B_i_) Schematic illustration of the synthesis procedure, (B_ii_) visual observation of resulting flakes on a grid (0.9 × 0.9 cm^2^), (B_iii_) optical microscopy images, and (B_iv_) scanning electron microscopy (SEM) image. Panel B reprinted with permission from Zhou et al. [49]. Copyright 2015, WILEY‐VCH Verlag GmbH & Co. KGaA, Weinheim. (C) Chemical vapor deposition‐based gold flake growth. (C_a_) Schematic of the experimental setup. (C_b_) SEM image of the resultant gold microplates. (C_c_) PL spectrum of a single gold microplate. Panel C Reproduced from Wang et al. [123], with permission from the Royal Society of Chemistry. (D) Combined top–down and subsequent bottom–up approach for gold flake arrays synthesis. (D_a_) SEM image of a periodic array of gold nanostructures acting as seeds for flake growth. (D_b_) Schematic representation of the solution‐based growth mode. (D_c_)‐(D_d_) SEM images of the resulting flake array. Reprinted with permission from Demille et al. [106]. Copyright 2021, Tsinghua University Press and Springer‐Verlag GmbH Germany, part of Springer Nature.

Growth can proceed in a template‐free colloidal environment [67, 109], or through templated approaches using rigid structures, such as ITO [110], and graphene [111], or soft structures, including polymers [112], and even protein fibers [49]. Additionally, capping agents that selectively adsorb onto basal planes, along with stabilizers and other interfacial components, play a crucial role in controlling morphology and preventing aggregation [31, 74, 113]. Selected examples of flake synthesis approaches are presented in Figures 4 and 5. For instance, Farkas et al. [114], employed a classical wet‐chemical reduction method within a gel matrix, creating a gradient of reducing agents across the medium. This approach yielded flakes of varying sizes, spatially organized within the gel (Figure 4A). Lv et al. [60], and Krauss et al. (now Schatz et al*.)* [76], adopted template‐directed synthesis routes, with the latter utilizing rigid glass surfaces as templates (Figure 4B), whereas the former demonstrated a bio‐inspired approach using filamentous proteins that served as both a structural template and a reducing agent (Figure 4C). The resulting flakes reach macroscopic size, up to millimeters in lateral dimensions, with thicknesses ranging from 0.4 to 1.3 µm, visible to the naked eye [49, 60].

In wet chemical synthesis, there appears to be a lower limit to the aspect ratio (i.e., the ratio of thickness to lateral area). For large‐area flakes, the minimal achievable thickness is typically around 10 nm. If this thickness is still too large for a particular application, it can be further reduced through thinning or etching procedures [37] (Figure 4D).

An alternative way to categorize gold flake synthesis is based on procedural frameworks, such as single‐step seedless approaches [6, 76], multi‐step seed‐mediated synthesis [64, 115, 116, 117], or distinctions between template‐directed [49, 51, 66], and template‐free strategies [58], surfactant‐assisted methods [35, 118], polymer‐assisted synthesis [106, 114], liquid crystal‐based approaches [82, 119], and on‐substrate fabrication techniques [66, 110, 120], among other classifications and variations [58].

Among chemical synthesis techniques, polyol‐based wet‐chemical synthesis [2, 121, 122], and its modifications have emerged as the most widely employed methods for producing gold flakes [31, 66, 76, 81, 102, 110]. In this method, a gold precursor is reduced in a polyol medium, typically ethylene glycol, which serves as both solvent and weak reducing agent. Alternatively, external reducing agents such as citrate or aniline may be added [67, 124]. The final flake morphology is governed by reaction parameters, including precursor concentration, temperature, reaction time, pH, and the presence of surfactants or capping agents [31, 125]. Commonly used additives such as PVP and cetyltrimethylammonium bromide (CTAB) serve as stabilizers or surfactants [81, 126]. They are believed to selectively bind to specific crystallographic facets, directing anisotropic growth while maintaining colloidal stability and preventing aggregation [2, 34].

Beyond PVP and CTAB, a diverse range of molecular species including other surfactants [125, 126], polymers [51, 126], biomolecules [49, 127], small organic molecules [128, 129], adsorbed gases [105, 123], and even atomic species such as metal ions [8, 66, 78], have been explored to fine‐tune flake growth. The careful selection and combination of these components enable precise control over size and shape evolution [74, 78, 113, 130]. For instance, Qin et al. [35], demonstrated the tunability of flake thickness by adjusting the ratio between gold precursor ions and the directing surfactant that reduces them (Figure 5A). These additives can be introduced during the initiation of the metal reduction or continuously throughout the reaction [64, 115, 116, 117], underscoring the complex interplay between reaction components and their concentration in space and time in defining flake morphology.

Biological synthesis has emerged as a promising method, leveraging biomolecules and molecular biological elements such as microorganisms, viruses, plants, proteins, and DNA molecules as templates, stabilizers, or reducing agents [75]. The first biosynthesized gold flakes exceeding 0.5 µm were reported by Shankar and colleagues [52, 131], paving the way for subsequent studies that refined the synthesis process using various biomolecules. These include amino acids [128, 129], plant‐derived molecules [52, 132], polysaccharides [133, 134], and fungal‐based extracts [135].

With later technological applications in mind, the biological or so‐called “green” methods offer an environmentally friendly, biodegradable, and biocompatible synthesis alternative, avoiding residues of toxic synthesis components in the final gold flake ensemble [75]. These sustainable approaches eliminate the need for organic solvents, harsh chemicals, stabilizers, conventional surfactants, and toxic polymers or crosslinkers, replacing them with benign biomaterials, instead, significantly reducing environmental toxicity and biological hazards. For example, Zhou et al. [49], demonstrated the synthesis of macroscopic gold flakes under mild, green conditions by utilizing amyloid fibril proteins, which serve multifunctional roles as reducing, directing, and stabilizing agents (Figure 5B).

Physical flake synthesis approaches are less common. They incorporate physical tools to drive the reduction process by supplying the necessary energy and/or electrons. This includes vapor techniques [123, 136], typically employing thermolysis and a carrier gas system (Figure 5C), photoreduction, where photons facilitate electron transfer [55], electroreduction, which utilizes an electrode as an electron source [137], as well as microwave‐assisted synthesis [138]. Finally, there are approaches combining multiple techniques, including classical fabrication methods. For example, predetermining the flake positions by nano imprint lithography and subsequent seed placement by a sophisticated single crystallite synthesis yielded arrays of flakes (Figure 5D).

A more versatile strategy for deterministic placement of a single gold flake relies on preparing an ensemble of flakes with varying diameters and thicknesses by synthesis on a well‐defined substrate. Subsequently, a flake exhibiting the desired geometrical and optical properties is selected and transferred to the target location using a polymer‐assisted transfer technique [76, 139], a method commonly handy for rapid prototyping. A broader overview of transfer methods for two‐dimensional materials is available in the recent review by Cheliotis et al. [140].

## 5 Applications

All gold flakes exhibit distinct chemical and physical properties that make them advantageous for a wide range of applications [31]. However, in cases where conventional synthesis of simple geometries such as triangular, spherical, or hexagonal particles results in limited surface area and restricted geometric flexibility [141, 142, 143], large‐area flakes become essential [144, 145, 146, 147, 148, 149]. In such contexts, the ability to structure a material laterally with nanometer‐scale precision and strictly controlled thickness while maintaining large‐area single crystallinity is critical. These attributes are particularly important in nanophotonics and plasmonics, where gold flakes serve both as versatile substrates for top–down fabrication of complex nanostructures and as functional components in standalone devices.

The physicochemical properties of gold flakes are intrinsically linked to their size, shape, and crystallographic structure, and can be tuned to meet specific application requirements through controlled synthesis or post‐synthesis structuring. A wide range of structural and surface parameters can be tailored through synthesis and post‐synthesis structuring, including edge length, thickness, surface functionalization, and edge morphology [1, 31, 61, 150, 151].

Owing to their unique properties, high‐aspect‐ratio gold flakes have been widely utilized across various fields. In the following, we will highlight a few of these, sorted by topic and backed up with more comprehensive tables. While most applications lie in photonics and related fields, gold flakes have also been utilized in nanoelectronics, medical diagnostics and sensing. In addition, these large flakes serve as building blocks for complex nanodevices, catalysis, and other emerging applications. An extensive overview of the available studies is compiled in structured tables within the relevant sections.

**TABLE 1:** Gold flake applications in nanophotonic

Method	Application summary	Refs.
As synthesized	Fundamental properties	[25, 83, 87, 158, 159, 160, 161, 162, 163, 164, 165, 166, 167, 168, 169, 170],
	Surface plasmons	[35, 157, 171, 172, 173, 174, 175, 176, 177, 178, 179, 180],
	Edge plasmons	[84],
	Quantum emitter enhancement	[144, 181],
	Strong vibrational coupling	[182],
	As substrate/mirror	[183, 184, 185, 186, 187, 188, 189],
	Thinning	[37],
	LSPR properties and control	[25, 161, 190, 191, 192, 193, 194],
	Brillouin scattering	[195],
	Nonlinear optics	[83, 87, 164, 196, 197],
Structured: resonators	Rods	[6, 147, 198, 199, 200, 201, 202, 203, 204, 205, 206, 207, 208, 209, 210],
	Slits	[211, 212, 213, 214, 215, 216, 217],
	Evolutionary optimized	[218],
	Electrically connected rods	[146, 149, 219, 220, 221, 222, 223, 224, 225, 226, 227],
	Other shapes	[228],
Structured: waveguides	Fundamental properties	[229, 230, 231, 232, 233, 234, 235, 236, 237, 238],
	Nonlinear optics	[162, 239, 240],
	Quantum emitter enhancement	[212, 216, 241, 242],
Structured: full devices	Receiver and emitter	[243],
	Quantum emitter enhancement	[244],
	Plasmon logic	[245, 246],
Structured: gratings/meta surfaces	Fundamentals	[60, 148, 247, 248, 249],
	Focusing	[156, 250],
	Sensing	[251],
	Nonlinear	[252, 253, 254, 255],
	Photoemission	[256, 257],
Flake hybrids	Phonon–polaritons	[258, 259, 260, 261, 262],
	Non‐locality	[97],

*Note*: “As synthesized” means gold flake without further fabrication or modification.

**TABLE 2:** Au flakes as electronic building blocks

Method	Application summary	Refs.
As synthesized	Building block in electrocatalytic electrodes	[105, 276, 277, 278, 279],
	Electrical circuit building blocks	[10],
	Flexible device for resistive switching	[273],
	Nanocomposite with tunable conduction	[271],
	Electrically conductive coatings for vapor sensing	[132, 280],
	Gold flake‐based aerogel to conductance pressure sensor	[275],
	Chitin nanofiber hybrid film sensor for humidity, pressure, and more.	[272, 279],
	Strain control electrical devices	[49],
Functionalized flake	Electrical circuit building blocks	[9, 274, 281, 282],
Structured	Open‐circuit photovoltaic device	[283],

*Note*: “As synthesized” means gold flake without further fabrication or modification.

**TABLE 3:** Integrating Au flakes into sensing platforms.

Method	Application summary	Refs.
As synthesized	Drop‐casting and drying for SERS substrate usage	[289, 290],
	Synthesized and embedded within a nanofibril matrix	[291],
	Within the CNT sheet flexible matrix for SERS	[292],
	Within the PDMS elastomer matrix for SERS	[301],
	Possible NIR‐absorbing or antennas for hyperthermia of cancer cells	[131],
	Optical plasmonic change of Au flake upon reaction with mercury	[287],
	Viscosity sensing of liquids	[302],
	Sensing of H_2_O_2_	[303],
Functionalized flakes	Functionalized with antibodies for selective SERS sensing	[294, 298],
	Functionalized with DNA for selective electrochemical biosensing	[304],
	Folic acid conjugated for cancer detection	[293],
	Synthesizing nanotip structures on the flake surface for SERS	[60],
	Gold nanoparticles were grown on the flake surface for SERS	[295],
	AgNCs deposition on the surface of Au flake for SERS sensing	[288],
Structured	PDG sensing platform for: environmental index, thickness, hydrogen	[296, 305, 306],
	Evolutionary optimized SERS sensing platform	[36],
	AuNPs grown on the flake with ultrasound‐induced hollow gaps	[297],
	Flakes with multiple cracks for SERS	[307],

*Note*: “As synthesized” means gold flake without further fabrication or modification

**TABLE 4:** Gold flakes for scanning probe microscopy.

Method	Application summary	Refs.
As synthesized	Substrate for TERS investigating physical absorbent materials	[316, 330],
	TERS characterizing graphene‐like and graphitic sheets	[318],
	Substrate for STM to probe radiative electromagnetic LDOS	[319],
Functionalized flakes	Flat substrate for STM sample investigation	[309],
	TERS‐based SAM analysis	[311],
	TERS‐based reaction analysis	[331, 332],
	Immobilization substrate for TERS biomolecule investigation	[312],
	Study of thiolated molecule species with polarized light	[317],
	Quantitative information of the SPR and NF temp. by TERS	[313],
	Electrical tunneling current imaging	[310],
Structured	Scanning probe resonantly coupled to a colloidal quantum dot	[212, 216],

*Note*: “As synthesized” means gold flake without further fabrication or modification.

**TABLE 5:** Gold flakes as a catalytic platform.

Method	Application summary	Refs.
As synthesized	Graphene – Au flakes hybrid for high catalytic activity	[324],
	Plasmonic catalysis on a flake	[331, 332],
	Electrochemical catalytic oxidation of analytes by gold flakes on ITO	[276],
	Catalytic degradation of Azo compounds using NaBH_4_	[333],
	Catalytic hydrogenation of furfural, and H_2_O_2_ catalytic decomposition	[293],
	Catalytic electrooxidation of formic acid/methanol	[105, 277, 278],
	Catalysis of 4‐nitrophenol	[303, 338],
	Electrochemical catalytic activity of H_2_O_2_	[279],
	Catalytic activity mapping	[339],
	Bimetallic nanoplates catalysis	[327, 328, 340],
Structured	Oxidation/reduction reactions of Fe(CN)_6_^4−^/Fe(CN)_6_^3−^	[334, 341],

*Note*: “As synthesized” means gold flake without further fabrication or modification

**TABLE 6:** Gold flakes in other applications.

Method	Application summary	Refs.
As synthesized	Miniature capacitive picobalances	[335, 336],
	Fluorescent enhancement for cell studies	[342],
	Thermoplasmonic platform for assembly of colloids	[353],
	Epitaxial Growth	[48, 315, 346, 347, 348, 349, 350, 351, 352],
	Tuning the thermal properties of hybrid materials	[291],
	Printing and pigment application	[362],
	Perfect mirror for Casimir‐forces	[360, 368, 369, 370, 371],
	Optomechanical force	[356, 357, 358, 359],
Structured	Plasmonic antenna to mechanically anchor organic molecules	[343],
	Light‐driven microdrones	[354, 355],
	FIB/EBL‐based versatile nano‐building blocks	[344, 345],
Functionalized flakes	Drug delivery and phagocytosis analysis	[337],

*Note*: “As synthesized” means gold flake without further fabrication or modification.

### 5.1 Photonics and Plasmonics

The well‐known reddish color of nanometer‐sized gold particles arises from localized surface plasmon resonance (LSPR) [152], and gradually shifts to the infrared as the material transitions toward larger dimensions, where large‐area flakes exhibit the characteristic golden‐yellow color of bulk gold. These optical properties can be fully described by classical dielectric constant models such as the Drude‐Lorentz model [153]. Gold flakes exhibit close to perfect crystallinity, maximizing conductivity and reducing heat generation of LSPRs due to ohmic losses to the theoretical minimum [6, 36, 37]. Their large contact area with any surface is, in general, an advantage, as homogeneously grown “normal” single gold crystals would be roughly spherical and, therefore, have only a small and difficult to predict contact area with a substrate. Finally, macroscopic crystals are too extended perpendicular to the substrate to be conveniently further structured by standard top–down techniques (in one exception, one has been used for creating plasmonic ridges on top of the crystal [154]). Consequently, gold flakes have been used in a plethora of optics and photonics‐related research, as summarized in Table 1.

Pristine, unstructured gold flakes provide a chemically stable, well‐defined environment for investigating fundamental properties of surface plasmon polaritons (SPPs) in 1D (at the edges) and 2D (on the surface). SPPs form when electromagnetic waves with frequencies smaller than, but near a metal's plasma frequency (ω_*p*_ (*Gold*) =  13, 8 · 10^15^ Hz) interact with the metals' conduction band electrons, yielding a coupled state between the quasi‐free electrons and light [155]. The currents driven with these high frequencies are prone to ohmic losses, in contrast to metals being perfect electrical conductors for low frequencies. To effectively apply plasmons (a word often used instead of SPP), any additional losses due to crystal faults should be kept minimal, an intrinsic feature of monocrystalline gold flakes. Plasmon propagation phenomena have been researched extensively [84, 156, 157], and are a possible method to measure the dielectric function of monocrystalline gold. As gold flakes can be transferred, e.g., via polymer droplet methods [139], the quantum phenomenon of non‐locality has also been examined by stacking flakes with extremely thin spacers made from 2D materials [97].

For more sophisticated applications, gold flakes offer an exceptionally well‐suited starting substrate for top–down fabrication of well‐defined complex geometries via lithographic and/or milling techniques [7, 36, 146, 147, 148, 149]. Desired functional elements contain optical antennas, plasmonic waveguides for nanocircuitry systems, and more advanced and hybrid structures integrating multiple optical and optoelectronic components [25, 37, 145, 146, 149, 163, 166, 182, 249, 258].

**FIGURE 6:**
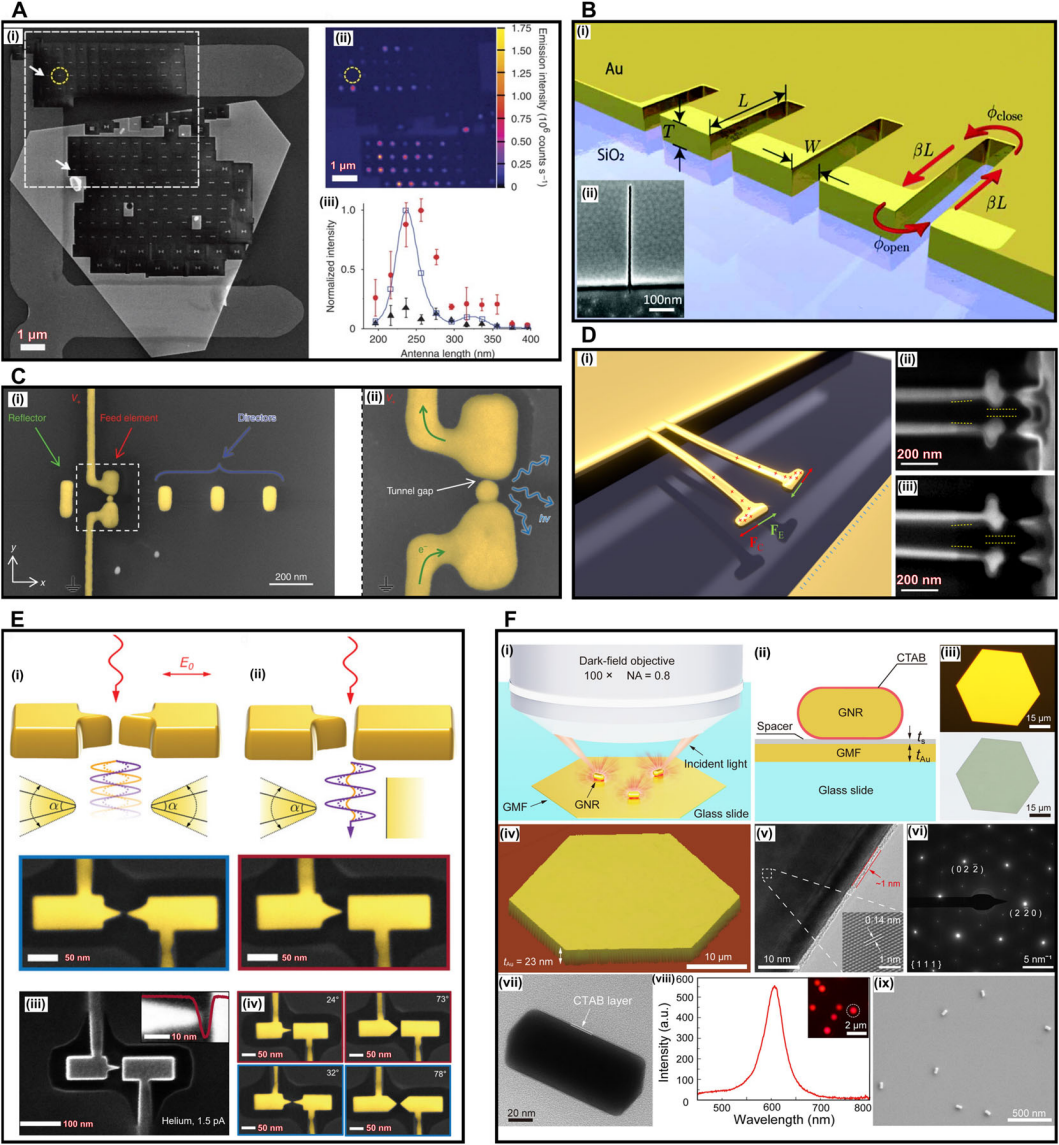
Applications of optical antennas made from monocrystalline gold flakes. (A) Bowtie and linear antennas fabricated in both monocrystalline and polycrystalline gold. (A_i_) SEM image of linear antennas fabricated from monocrystalline gold flake (middle) with patches of a vapor‐deposited polycrystalline gold film (upper portion), with the respective two‐photon photo luminescence mapping (TPPL) in (A_ii_). In (A_iii_), the averaged integrated TPPL intensity from linear nanoantennas on monocrystalline (red dots) and polycrystalline (black triangles) gold. Panel A reprinted with permission from Huang et al. ref. [6]. Copyright 2010, Springer Nature Limited. Panel B shows plasmonic nano slit antennas fabricated by helium ion milling. (B_i_) Schematic visualization with corresponding SEM at (B_ii_). Panel B Reproduced from Chen et al. [211], with permission from the Royal Society of Chemistry. (C) Plasmonic Yagi‐Uda antenna driven by inelastic electron tunneling. (C_i_) SEM image of the antenna containing a reflector, a feed element with kinked connectors, and three directors fabricated via Ga‐FIB on glass. A magnified view of the feed element in (C_ii_) (dashed white rectangle) reveals an asymmetrically positioned particle attached via dielectrophoresis, forming a tunnel gap toward the top antenna arm. Panel C adapted from ref. [149]. Copyright 2020, The Author(s). (D) An electromechanically tunable suspended nanoantenna. A 3D schematic of the structure is depicted in (D_i_), while SEM images in (D_ii_) and (D_iii_) illustrate how the gap width expands from 40 nm to 70 nm as the applied voltage increases from 0 V to 20 V. Panel D Reprinted (adapted) with permission from Chen et al. [146]. Copyright 2016, American Chemical Society. (E) Effect of local symmetry breaking on the second harmonic generation (SHG) process in gold nanoantennas. Upon excitation with a linearly polarized laser at frequency *ω*, the SH efficiency depends on the gap geometry. (E_i_)‐(E_ii_) Symmetric‐gap antenna and asymmetric‐gap antenna, respectively, with colored SEM images. Both antennas have a gap size of 9 nm. (E_iii_) shows an example of the minimum achievable gap size of < 5 nm. The red line displays the line profile along the center of the SEM image. (E_iv_) tuning SHG by varying the degree of local symmetry breaking. Panel E reprinted from Meier et al. [264]. Copyright 2023, The Authors. Advanced Optical Materials published by Wiley‐VCH GmbH. (F) highlights the integration of gold nanorods (GNRs) with gold micro flakes (GMFs). (F_i_) and (F_ii_) provide a schematic visualization of the design and its cross‐section, while (F_iii_) presents optical microscopy images in both reflected and transmitted modes. The structural characteristics of the gold flake are further detailed through atomic force microscopy in (F_iv_) and TEM imaging in (F_v_), which includes a high‐resolution zoom‐in and the corresponding electron diffraction pattern shown in (F_vi_). In (F_vii_), a TEM image of a GNR. (F_vii_) Scattering spectrum of a GNR on a glass. Inset: dark‐field scattering image of the measured GNR (circled). (F_ix_) shows a SEM image of the assembled system of GNR on a gold flake. Panel F Reprinted (adapted) with permission from Liu et al. [183]. Copyright 2022, American Chemical Society.

Structured monocrystalline large‐area gold flakes were first reported as an optical material by Wiley et al. [198], where a mechanical skiving method was used to cut a flake into wires working as quasi 1D Fabry‐Perot resonators, where plasmons form modes comparable to a guitar string [263]. However, the first use of gold flakes as a basis for fabricating geometrically versatile plasmonic resonators and waveguides was reported by Huang et al. [6], (Figure 6). Here, e.g., double wire and bow‐tie shaped optical antennas were fabricated via Gallium based focused ion beam (Ga FIB) milling, realizing antenna gaps of only a few tens of nanometers where light is concentrated to volumes much smaller than the diffraction limit with several orders of magnitude higher intensities than possible with classical optics, verified via nonlinear two photon photoluminescence (TPPL). The superior optical properties of gold flakes were demonstrated in direct comparison to identical geometries made from sputtered gold (Figure 6A_iii_).

Optically accessible plasmonic resonances can also form in slits carved into a gold flake's edge [211]. With single‐digit nanometer widths fabricated by means of helium‐based focused ion beam (He FIB) milling, structures with multiple overlapping resonances are realized (Figure 6B), allowing for quantum properties of localized plasmons to be assessed.

The structural properties of gold flakes also allow the realization of long, smooth wires using ion‐beam milling [6]. This allows for electrically connecting plasmonic optical antennas with undisturbed plasmonic resonances [219]. Based on this, Kern et al. [220], used dielectrophoresis to trap a nanoparticle in the antenna gap, allowing the excitation of the plasmon resonance via inelastic electron tunneling, finally leading to spectrally shaped light emission. As a follow‐up development, Kullock et al. [149], demonstrated highly directive, compact, and electrically driven Yagi‐Uda antennas (Figure 6C), offering a potential low‐footprint solution for chip‐based photonic communication applications. Another idea has been shown by Chen et al. [146], suspending a connected optical nanoantenna in air so that the gap between the antenna arms can be expanded by charging the antenna arms, as shown in Figure 6D. This electromechanically tunable device has possible applications in optical nanoelectromechanical systems (NEMS).

Pushing light localization to the extreme, He FIB allows to realize asymmetric gaps smaller than 5 nm with tip radii as low as 8 nm (Figure 6E) to optimize nonlinear optical effects like second harmonic generation (SHG). Smaller gaps can be realized by (nano) particle on a mirror ((n)POM) designs [265], where gold flakes serve as the optimal mirror for the charges of the particles LSPR [183, 184, 187]. This configuration facilitates extreme optical confinement in a thin layer between the flake and the nanoparticle (Figure 6F) [183]. Similarly, silver nanoparticles on gold flakes have been used [184].

**FIGURE 7:**
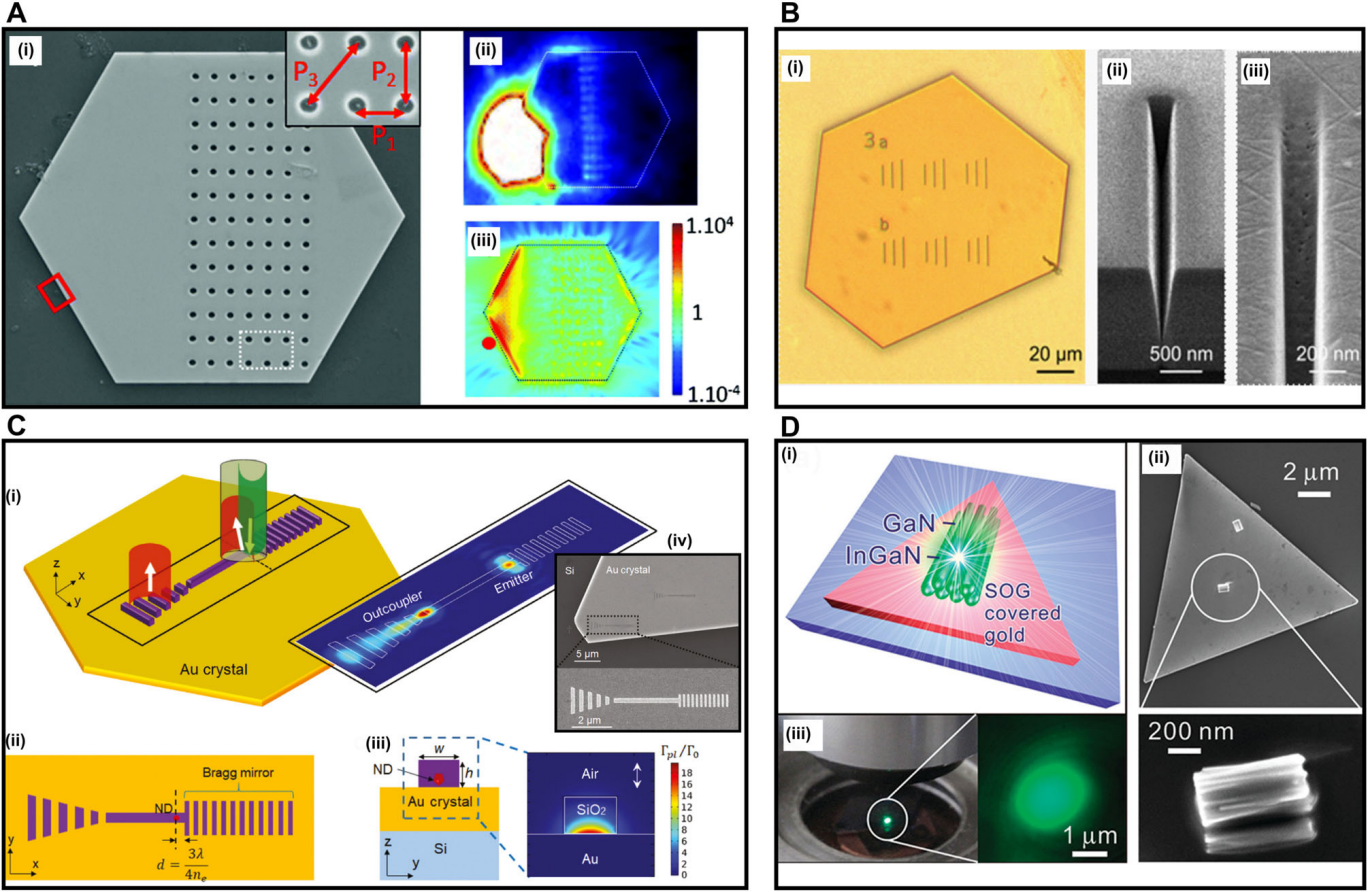
Monocrystalline gold flakes for light–matter interaction. (A) Plasmonic hole array fabricated *via* FIB milling on a monocrystalline gold flake to investigate plasmon propagation excited by a single photon emitter. (A_i_) SEM image of the milled structure, with the red rectangle indicating the quantum nano emitter's location and highlighting key geometrical parameters corresponding to the white dashed rectangle. (A_ii_) Experimental and (A_iii_) simulated surface plasmon signal transfer maps on the gold flake surface. Panel A reproduced from Kumar et al. [181], with permission from the Royal Society of Chemistry (B) Plasmon polariton channel's structure to couple to single fluorescent molecules. (B_i_) shows a microscope image of a gold flake containing V‐grooves of varying sizes. (B_ii_) Helium ion microscopy image showing a cross‐section of a V‐groove alongside a nano mirror. (B_iii_) Zoomed‐in view of the nano mirror at one end of the V‐groove. Panel B reprinted (adapted) with permission from Kumar et al. [242]. Copyright 2020, American Chemical Society. (C) Single‐photon emitter platform based on a structured dielectric on top of a flake. (C_i_) and (C_ii_) represent a schematic of the device layout and working principle. Dielectric nano ridges were fabricated by EBL atop a gold flake. The simulated far‐field image for the coupled system is shown on the right of (C_i_). (C_iii_) Cross‐section visualization of the system, including mode profile, indicating the distribution of Purcell enhancement. (C_iv_) SEM image of the fabricated device on a gold crystal. Panel C adapted from Siampour et al. [244]. (D) Gold flake‐based green nano laser (spaser). (D_i_) Schematic illustration of the lasing architecture, comprising a bundle of green‐emitting semiconductor nanorods coupled to an underlying monocrystalline gold flake. (D_ii_) FE‐SEM image of the hybrid system, with a magnified view detailing the InGaN/GaN nanorod bundle positioned atop the gold flake. (D_iii_) Emission of green laser light from the hybrid structure. Reprinted (adapted) from Wu et al. [258]. Copyright 2011, American Chemical Society.

The possibility of achieving a large field enhancement near plasmonic resonators is identical to providing an increased local density of states (LDOS) at the respective position, which enhances the emission rate of excited electronic states [266]. Also known as “Purcell enhancement,” there is a classical derivation which relates emission enhancement to large quality factors and small mode volumes of a resonator [267]. Therefore, structures made from monocrystalline gold surpass polycrystalline materials, as both the q‐factor and the quantum efficiency, the ratio of originally emitted photons reaching the far field, increase proportionally to the conductivity of the resonator material. For example, the photoluminescence (PL) of single quantum dots coupled to a gold flake in dependence on the thickness of a PMMA spacer layer shows a significant change in the optical properties [144]. Examples of single emitters coupled to (structured) gold flakes are displayed in Figure 7. Also termed hybrid optical antenna structures, they have been realized using fluorescent molecules, quantum dots, or defects in 2D/3D materials.

Coupling can be achieved by placing the single emitter at the edge of a flake, as realized in [181], (Figure 7A), using a nano diamond with a single nitrogen‐vacancy center. In addition, a grating was engraved into the flake, acting as a plasmon wavelength filter. An example of a molecular fluorescent photon source is dibenzoterrylene (DBT) molecules in anthracene nanocrystals. They have been integrated within grooves carved by FIB into gold flakes (Figure 7B) [242], showing a 50% emission enhancement and 14 µm plasmon decay length. More sophisticated geometries employ antennas and waveguides toward realizing a full nanophotonic device. A nanostructured dielectric material on top of a gold flake can be designed to realize waveguiding modes, Bragg mirrors, and optical antennas [244], (Figure 7C). This allows for structuring around an embedded single emitter, e.g., a nano diamond containing a GeV‐(Germanium‐vacancy)‐center as a single photon source that is enhanced 15‐fold.

A potentially disruptive application of plasmon hybrid devices is spasing [268], which is the abbreviation for Surface Plasmon Amplification by Stimulated Emission of Radiation. Sub‐diffraction‐limited laser operation in the green spectral region has been demonstrated in a hybrid metal‐oxide‐semiconductor (MOS) plasmonic nanocavity structure [258], (Figure 7D), again benefiting greatly from the low losses within gold flakes by minimizing the laser threshold. Additionally, monocrystalline gold‐based metasurfaces enabled precise control over the anisotropic and isotropic contributions to second harmonic generation (SHG) near localized surface plasmon resonance conditions [164, 255], making them valuable for studying nonlinear optical phenomena in metal thin films [83, 87], as well as nanophotonic imaging and probing of low‐dimensional materials [259].

**FIGURE 8:**
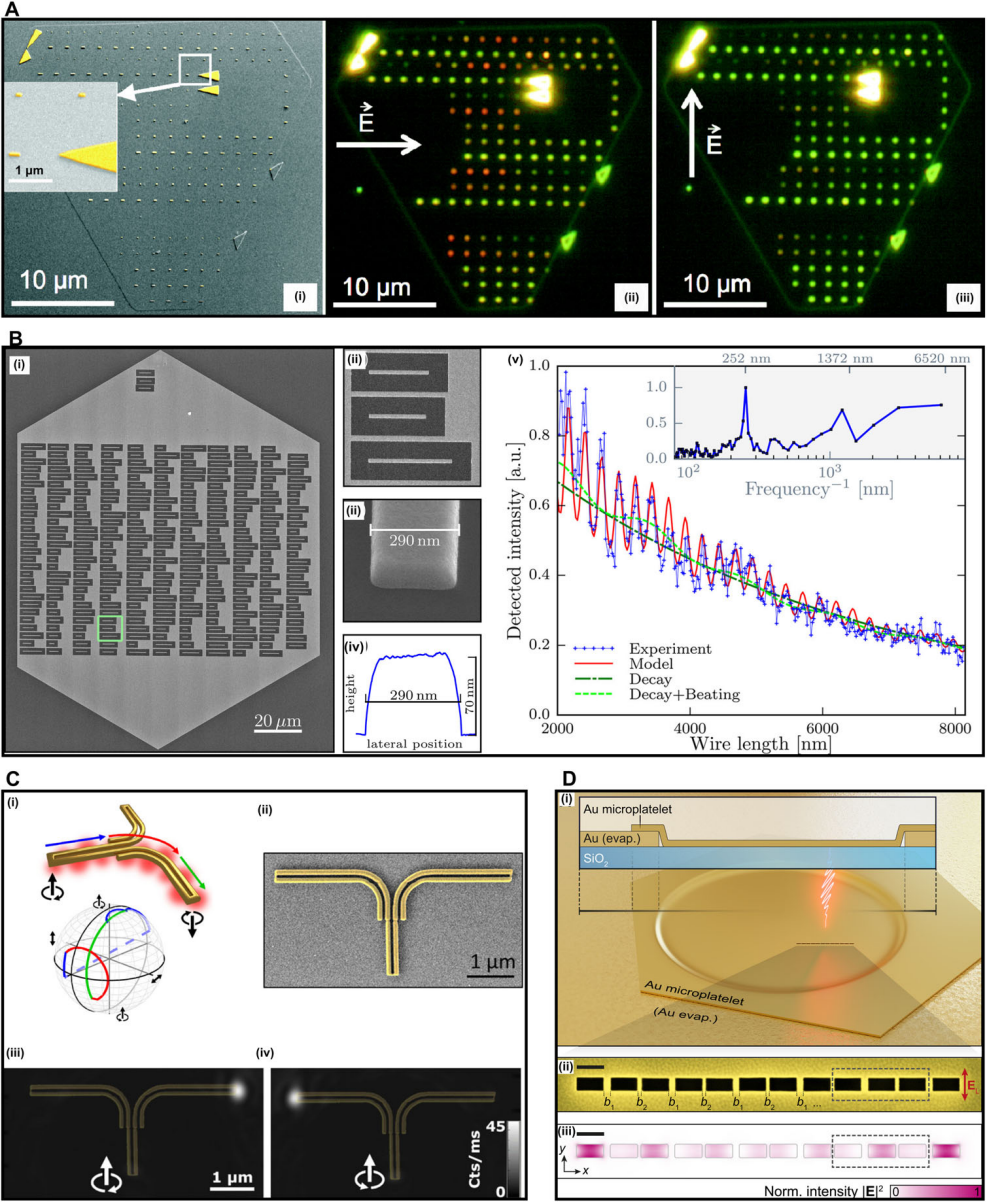
Non‐FIB fabrication and plasmonic circuits. (A) Shows a fabricated monocrystalline nanoantenna multiple arrays. (A_i_) presents an SEM image of nanoantenna rods sculpted from a large monocrystalline gold flake, with the inset providing a magnified view. (A_ii_) and (A_iii_) display dark‐field images captured under polarization aligned longitudinally and transversely to the rods, respectively. In (A_ii_), the color variations indicate a spectral redshift in the plasmonic resonance as the antenna length increases. Conversely, in (A_iii_), the color remains uniformly distributed, as the transverse polarization interacts with the short axis of the nanorods, which exhibits minimal variation across the array. The bright triangular features serve as alignment landmarks. Panel A adapted from MÉJARD et al. ref. [147]. Copyright 2017, Optical Society of America. (B) Fabricated plasmonic circuitry arrays for plasmon transmission analytical analysis. (B_i_) SEM image of the full fabricated arrays within a gold flake, with higher‐magnification views shown in (B_ii_) and (B_iii_). Panel (B_iv_) shows AFM profile of the wire cross‐section. (B_v_) demonstrate the detected signals from the plasmonic circuitry arrays, presented alongside the proposed Fabry–Pérot model. Reprinted (adapted) with permission from Geisler et al. [235]. Copyright 2017, American Chemical Society. (C) Spin‐Optical Nanodevice. (C_i_) Sketch of the spin‐optical nanodevice, where plasmons first propagate linearly (blue), then follow a curved path (red), and finally continue with another linear segment (green). The device functions similarly to an electron spin transistor, with in‐coupling (source), out‐coupling (drain), and a central gate region. In this gate region, the spin state is defined by the accumulated phase, acting like a gate voltage. The lower part shows the trajectory of the photon's pseudospin state throughout the device, represented on a Poincaré sphere. (C_ii_) colored SEM‐image. (C_iii_) and (C_iv_) show a CCD image for excitation of the left‐handed (C_iii_) or right‐handed (C_iv_) pseudospin state with the analyzer in the detection path set to transmit only emission of the left‐handed or right‐handed pseudospin state. The structure's position is indicated by the overlaid colored SEM image. Reprinted (adapted) with permission from Krauss et al. [237]. Copyright 2019, American Chemical Society. (D) Plasmonic nano slit Su–Schrieffer–Heeger (SSH) chain with nontrivial topology: (Da), Chains of plasmonic nano slit resonators written into a monocrystalline Au micro flake using helium focused ion‐beam milling. The microflake covers a hole in a vapor‐deposited Au film residing directly on a smooth glass substrate. (Db), Top‐view scanning electron microscopy (SEM) image (false color) of a nontrivial nanoslit SSH chain consisting of twelve coupled resonators separated by bridges with alternating widths, *b1* and *b2*, as indicated. (Dc), Simulated near‐field intensity of a mid‐gap mode (COMSOL) exhibiting localized near‐field intensity at the two outermost nanoslits. Reprinted from Schurr et al. [270]. Copyright 2025, The American Association for the Advancement of Science.

Beyond FIB milling, various other methodologies have been used to fabricate nanostructures either within gold flakes or on their surfaces, including electron beam lithography (EBL), direct electron‐beam writing (DEBW), and nanoscale skiving with an ultramicrotome [145, 147, 198, 230, 233, 244]. To realize an array of optical antennas spanning a whole gold flake, Méjard et al. [147], employed standard EBL, followed by dry etching (Figure 8A), improving scalability compared to FIB milling.

Plasmonic circuits make use of the light localization in two dimensions but elongate the third dimension to realize wave guides with deep subwavelength cross‐sections. Also, the plasmon wavelength is (possibly much) smaller than the vacuum wavelength of light with a given frequency (λ_*p*_ < λ) [263], promising small footprint logical circuits harnessing the speed of light. Using the large area of a gold flake, the fabrication of a vast amount of single‐wire plasmonic waveguides with randomly changing but never repeating lengths (Figure 8B) has advanced the quantitative understanding of plasmon propagation [235]. The reproducibility of the wire cross sections and end cap geometry, as well as the always identical crystallinity, allowed us to reliably model the structures analytically as Fabry–Pérot resonators [269].

Based on former fundamental research [229], Krauss et al. [237], utilized gold flakes to fabricate a multimode plasmonic nanocircuit composed of forked two‐wire transmission lines that can be used to reversibly map and sort photon spin states at the nanoscale (Figure 8C). The device relies on a symmetric and antisymmetric eigenmode that interfere in carefully fabricated bent waveguides. This control over the spin angular momentum of light could pave the way for innovations in quantum information processing and spin‐based photonic technologies. Figure 8D shows instead a waveguide made from a chain of slit resonators, with their distances carefully tuned in a way that a topologically protected plasmonic edge state emerges from the interference of all reflections and transmissions that can be measured via photo‐electron emission microscopy (PEEM) [270].

Furthermore, gold flakes have been widely studied as a model system for exploring fundamental optical properties in monocrystalline thin films, including strong vibrational coupling in ultrahigh frequency plasmonic nano resonators [182], observing plasmonic skyrmion dynamics with deep subwavelength resolution [163], revealing quantum–mechanical effects in the luminescence emanating from thin monocrystalline gold flakes [166], and detecting the plasmon–polariton quantum wave packet [215].

### 5.2 Electronics

Owing to their high conductivity and low resistive losses, high‐aspect‐ratio gold flakes have recently been used in nanoelectronic devices, particularly in applications where conductivity serves directly as the sensing metric [37]. Their homogeneous crystallinity and well‐defined thickness enable fabrication with single‐nanometer precision to achieve the minimal possible geometrical footprint [37, 264]. This facilitates applications in high‐dielectric‐constant materials, printable electronics, capacitors, and electrodes.

**FIGURE 9:**
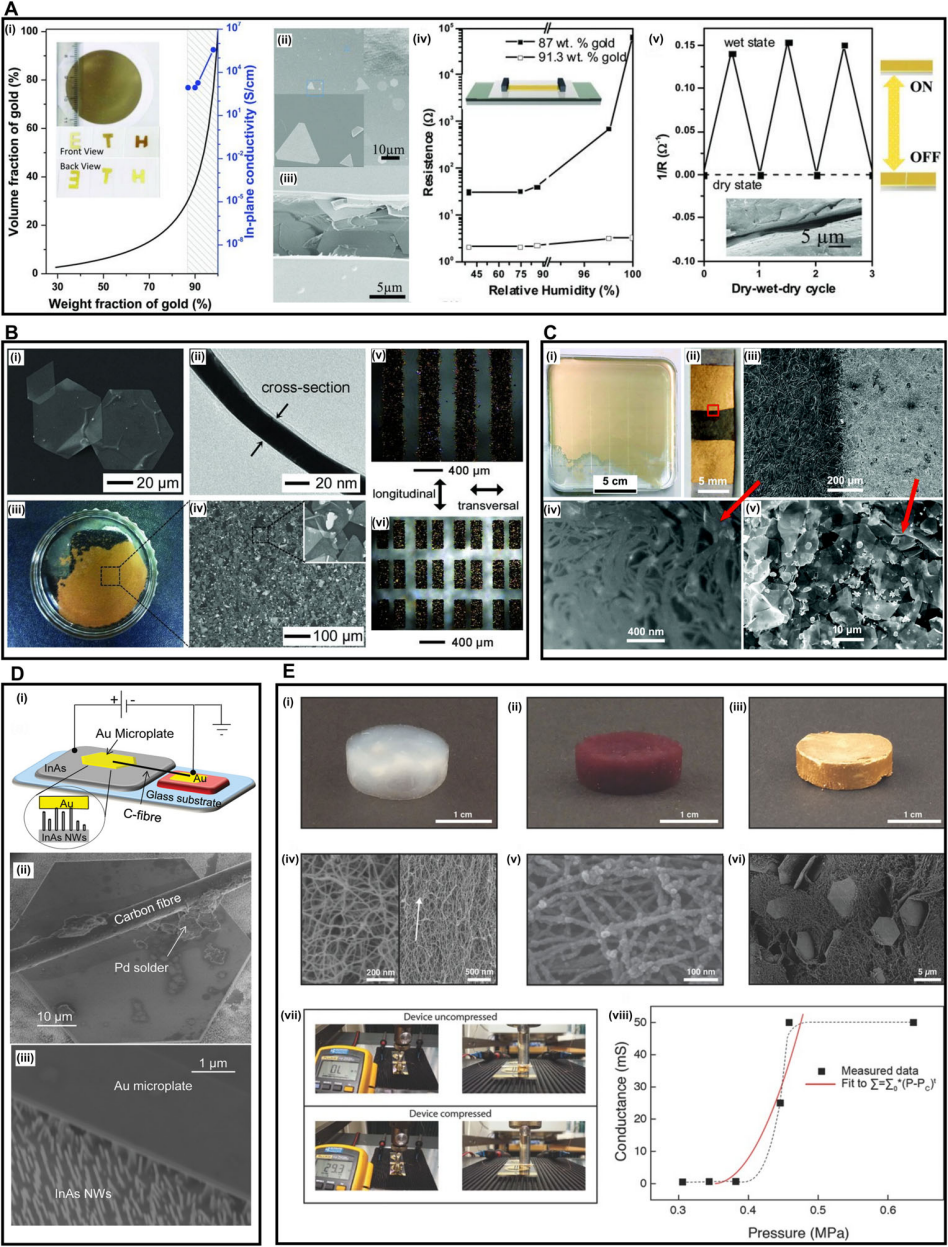
Monocrystalline gold flakes as electronic building blocks. (A) Applications of Gold Flake Amyloid Fibril Hybrid. (A_i_) Correlation between gold content in hybrid films and their conductivity. The inset shows a photograph of typical hybrid films. SEM images of the hybrid films depicting (A_ii_) surface morphology and (A_iii_) fracture section. (A_iv_) and (A_v_) Resistance‐based humidity sensor response for films with varying gold flake compositions. The first inset illustrates the device, the second a typical micro‐scratch. Panel A reprinted with permission from Li et al. [271]. Copyright 2013, WILEY‐VCH Verlag GmbH & Co. KGaA, Weinheim. (B) Stretchable patterned gold flake‐based electrode. (B_i_) SEM image of the synthesized gold flakes. (B_ii_) Cross‐section TEM image of a gold flake. (B_iii_) Gold flake film assembled on a water surface. (B_iv_) SEM image of the assembled Au flake film; the inset provides a magnified view showing overlapping Au flakes. (B_v_)‐(B_vi_) Optical microscopy images of patterned gold flake electrodes. Panel B reprinted with permission from Moon et al. [10]. Copyright 2013, WILEY‐VCH Verlag GmbH & Co. KGaA, Weinheim. (C) Preparation of a flexible gold flake‐based device. (C_i_) An assembled gold flake film on water. (C_ii_) Au flakes electrodes. (C_iii_) SEM image of the red square in (C_ii_). (C_iv_)‐(C_v_) The enlarged SEM images of electrode structures emphasizing the gold flakes in (C_v_) panel. Panel C Reproduced from Seo et al. [273], with permission from the Royal Society of Chemistry. (D) Gold micro flake device. Panel (D_i_) shows the configuration of the Au microplate position. (D_ii_) SEM image of the actual assembled device. The Pd metal between the carbon fiber and the microplate is also visible. (D_iii_) Zoom in on the nanowire structures beneath the gold flake along the edges. Panel D reprinted (adapted) with permission from Radha et al. [274]. Copyright 2012, American Chemical Society. (E) Gold flake hybrid aerogels. (E_i_)‐(E_iii_) Photographs of (E_i_) a standard aerogel, (E_ii_) a gold nanoparticle‐amyloid aerogel, and (E_iii_) a flake‐amyloid aerogel. (E_iv_)‐(E_vi_) SEM images of (E_iv_) the amyloid aerogel, (E_v_) the gold nanoparticle‐amyloid aerogel, and (E_vi_) the gold flake‐amyloid aerogel. (E_vii_) Photographs of a pressure sensor device incorporating gold flake‐based aerogels in its uncompressed (top) and compressed (bottom) states. (E_viii_) Conductance of the gold flake‐amyloid aerogel as a function of applied pressure. Panel E reprinted with permission from Nyström et al. [275]. Copyright 2016, WILEY‐VCH Verlag GmbH & Co. KGaA, Weinheim.

Li et al. [271, 272], introduced a hybrid nanocomposite of gold flakes and fibrils with tunable conductivity, ranging from insulating‐like behavior to values approaching pure gold conductivity that can be employed as a humidity sensor (Figure 9A). In a similar approach, gold flake–chitin nanofiber hybrids have been developed by Chen et al. [272], with possible applications in humidity sensing, breath analysis, and pressure sensing evaluation via electric conductivity of the hybrid circuit. Such a system could be used for speech recognition, health monitoring, or respiratory analysis, depending on its design and sensitivity.

Gold flakes have also been explored as contact and electrode materials in electronic circuits [10, 273, 274, 281, 283]. Moon et al. [10], proposed multilayered gold flakes as a novel stretchable electrode material suitable for organic‐based electronic devices (Figure 9B). These electrodes exhibited excellent electrical stability under repeated stretching cycles, highlighting their potential for printable and wearable sensing applications [29, 284]. Zhu et al. [283], reported a plasmonic platform based on nanowires fabricated from synthesized gold flakes that have been treated by electromigration to form irregular nanometer‐sized gaps for photovoltaic and electroluminescence applications. Seo et al. [273], demonstrated a flexible resistive switching memory device using an ultrathin composite film of gold flake nanosheets as electrodes (Figure 9C), highlighting its potential for wearable applications due to its paper‐like mechanical flexibility. Boya et al. [281], integrated and synthesized gold flakes as top contact electrodes in large‐area metal–molecule–metal electrode systems for the electrical analysis of molecules. Similarly, gold flakes were used to establish Ohmic top contacts for vertically grown nanowires of uneven height, leveraging electromigration of the (111) surface toward the shorter nanowires, as shown in Figure 9D [274].

In another example, biosynthesized gold triangles were assembled onto various substrates as building blocks in thin films for organic vapor sensing, where their resistance decreased upon exposure to weakly polar molecules [280]. This behavior suggests that an analyte containing molecular dipoles influences conductivity, enabling possible chemical sensing applications. Similarly, Ankamwar et al. [132], found that the polarity of an analyte plays a crucial role in determining film resistance. Nyström et al. [275], reported the development of ultralow‐density amyloid fibril‐based aerogels functionalized with gold flakes, which were employed as pressure sensors (Figure 9E) as their conductance varied as a function of the applied pressure. Finally, Zhang et al. [9], demonstrated the integration of gold flakes into electrical circuitry by functionalizing an Au triangle–chitosan matrix with an immobilized enzyme capable of glucose detection, monitored through changes in its cyclic voltammogram. Table 2 summarizes these and further gold flake applications in electronics.

### 5.3 Sensing

Also, in sensing applications, gold flakes excel due to low DC and AC ohmic losses, thereby improving the signal‐to‐noise ratio of any measurement. Their atomically smooth surfaces additionally ensure high device‐to‐device reproducibility through geometrically uniform fabrication, yielding quantitatively reliable signals contrary to stochastic substrates [36]. Their large areas allow for more sensitive surfaces when using grating resonances in addition to plasmonic effects [35]. Moreover, the surface readily supports functionalization via self‐assembled monolayers of thiolated molecules or antibodies specific to target analytes [150, 285, 286].

**FIGURE 10:**
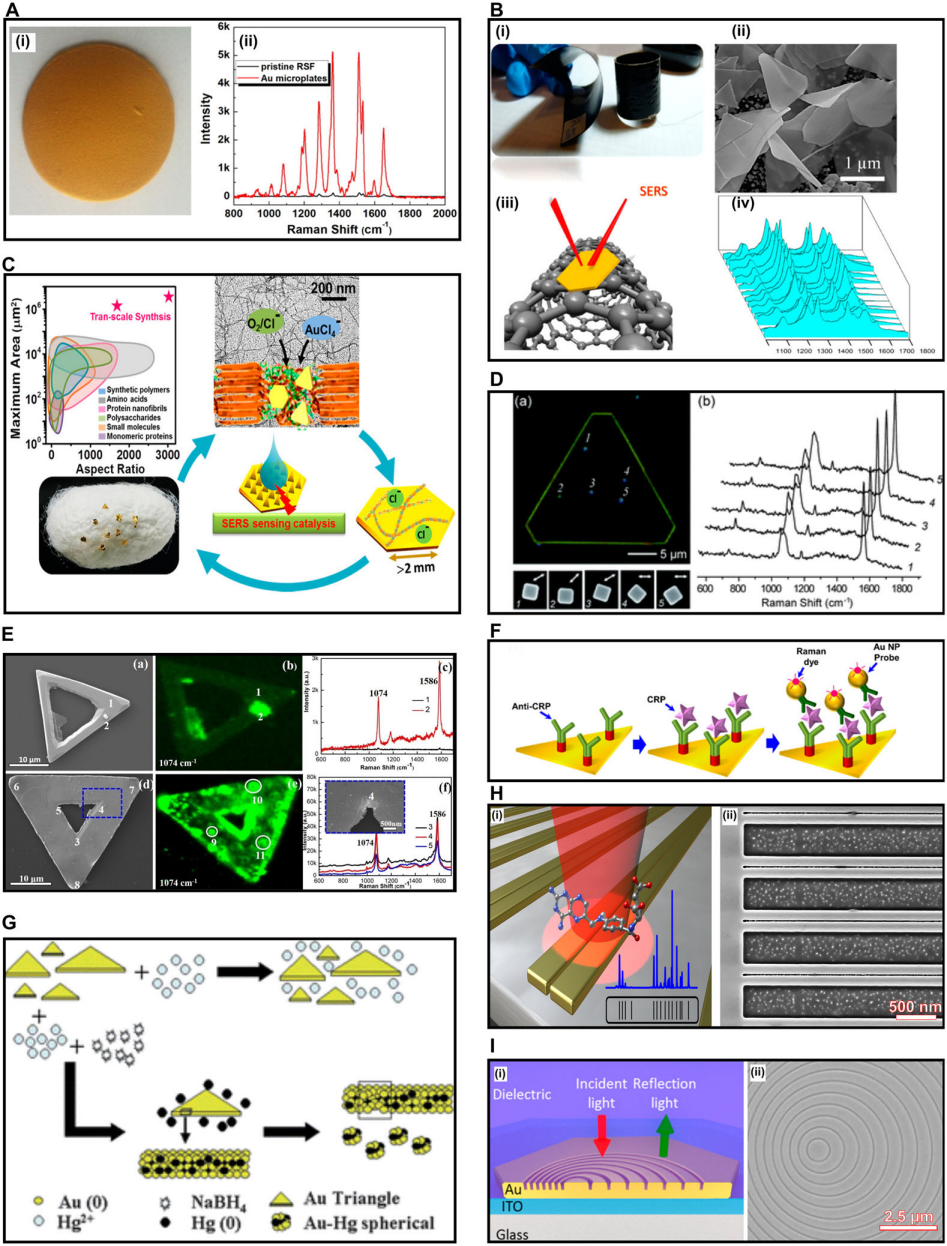
Monocrystalline gold flakes sensing applications. (A) Au flake hybrid material sensing: (A_i_) Photo of Au flake/silk nanofibrils composite. (A_ii_) Raman spectra of the analyte. Panel A reprinted from Fang et al. [291]. Copyright 2016, with permission from Elsevier B.V. All rights reserved. (B). Gold flake‐carbon nanotube (CNT) composite sensor. (B_i_) Image of a CNT sheet. (B_ii_) SEM of Au flake‐CNT sheet. (B_iii_) Schematic illustration of Raman signal enhancement. (B_iv_) Reproducibility evaluation of CNT sheet–Au flake as SERS substrate. Reprinted (adapted) with permission from Xin et al. [292]. Copyright 2017, American Chemical Soci*et*y. (C) Au flakes with lateral dimensions exceeding 2 mm for SERS applications, synthesized using natural fibrous proteins. Reprinted (adapted) with permission from Lv et al. [60]. Copyright 2018, American Chemical Society. (D) Gold flake‐silver nano cube sensing platform: (D_a_) Optical micrograph of five different silver nano cubes (AgNCs) on Au flake. (D_b_) SERS spectra recorded from the five AgNCs shown in (D_a_). Panel D Reproduced from Xia et al. [288], with permission from the Royal Society of Chemistry. (E) Hollow gold flake sensing platform: (E_a_), (E_d_) SEM images of the different platforms. (E_b_), (E_e_) Raman mapping of the analyte signal and corresponding plot shown in (E_c_), (E_f_). Reprinted from [297]. Copyright 2020, Springer‐Verlag GmbH Germany, part of Springer Nature. (F) C‐reactive protein (CRP) analyte gold flake immunosensor schematically illustrated. Reprinted (adapted) with permission from Hwang et al. [294]. Copyright 2019, American Chemical Society. (G) Gold flake‐based sensor for detecting femtomolar concentrations of mercuric ions, utilizing plasmonic changes induced by reaction with mercury. Panel G Reproduced from Singh et al. [287], with permission from the Royal Society of Chemistry. (H) Double resonator sensing platform: (H_i_)Geometry of the SERS sensing platforms. (H_ii_) SEM images of the FIB‐fabricated monocrystalline gold double gratings. Reprinted from Sweedan et al. [36]. Copyright 2024, The Authors. Small, published by Wiley‐VCH GmbH. (I) Plasmonic Doppler grating (PDG) index sensor. (I_i_) A schematic showing the structure of a PDG sensor and the configuration of the optical setup. (I_ii_) SEM image of a PDG fabricated on the surface of a monocrystalline gold flake for broad range index sensing. Reprinted (adapted) with permission from Lin et al. [296]. Copyright 2019, American Chemical Society.

This versatility of gold flakes facilitates their seamless integration into sensing and biomedical applications [9, 36, 60, 131, 132, 271, 272, 275, 279, 280, 287, 288, 289, 290, 291, 292, 293, 294, 295, 296, 297, 298]. For example, surface‐enhanced Raman scattering (SERS) is a powerful spectroscopic technique that amplifies Raman signals through localized surface plasmon resonances (LSPR) in noble metals, most notably gold [20, 299, 300]. Raman spectroscopy detects vibrational modes of solids and molecules via inelastic scattering of light, but with a factor of 10^6^ less efficiency than elastic Rayleigh scattering. However, since Raman signals scale non‐linearly with the local electromagnetic field, well‐designed plasmonic resonances enable highly sensitive molecular detection [299, 300]. Hotspots for SERS applications were created by leveraging the sharp tips and edges of triangular gold flakes, either on rigid flat substrates formed from dried drop‐cast films [289, 290], or through introducing Au flakes into hybrid nanocomposites. These included natural 3D matrices such as silk nanofibrils [291], chitin [272], and amyloid fibrils [275], which served as scaffolds for synthesis and immobilization, generating randomly scattered gold flakes within the matrices film, as shown in Figure 10A [291]. Similarly, synthetic 3D matrices, such as CNTs (Figure 10B) and polydimethylsiloxane (PDMS) elastomer, were employed to fabricate a flexible, gold flake‐based SERS substrate [292, 301], exhibiting superior SERS performance compared to its AuNP–CNT counterpart.

Gold flakes can also be utilized as platforms for post‐synthesis modification. For example, Lv et al. [60], reported huge gold flake synthesized biologically via silk fibroin (Figure 10C) as a platform to grow randomly scattered nanotips. LSPRs of these tips generate hotspots suitable for SERS applications, e.g., to monitor catalytic reactions. In an alternative approach reported by Xia et al. [288], silver nanocubes (AgNCs) were deposited onto a flake surface to fabricate a particle on mirror sensors for SERS (Figure 10D). Chen et al. [295], developed a large‐scale, two‐dimensional, flexible SERS‐based functional platform by growing gold nanoparticles (AuNPs) in situ on the flake surface. Furthermore, Wang et al. [297], advanced mono‐metal epitaxial growth by fabricating AuNP/Au flake hybrid structures featuring ultrasound‐induced hollow gaps within the flakes, significantly enhancing their potential for SERS applications (Figure 10E).

Functionalizing large‐area gold flakes with molecular recognition elements [308], enables the selective detection of specific analytes from complex mixtures, facilitating the isolation of the desired signal from unwanted background noise [294, 298]. Hwang et al. [294], reported a functional SERS‐biosensor for the trace detection of protein biomarkers. The platform was achieved by functionalizing the gold flake surface with specific recognition antibodies, thereby enhancing the sensor's specificity, as illustrated schematically in Figure 10F. Caño et al. [304], functionalized the flakes with dithiolated oligonucleotides that have been employed to develop an amplification‐free electrochemical biosensor for SARS‐CoV‐2 in patient samples. Similarly, gold flakes were successfully used to detect cancer cells in serum samples [293], macromolecules, including ovarian cancer biomarkers [298], as well as monoatomic ions, such as mercury, where optical monitoring after chemical reduction of the ions allowed detection of their concentration, as demonstrated by Singh et al. [287], (Figure 10G).

In addition, gold flakes can serve as versatile platforms for the development of novel structures. For example, Sweedan et al. [36], employed gold flakes to fabricate an evolutionarily optimized plasmonic sensor for SERS applications (Figure 10H), working with a wide range of analytes across different states of matter, including nucleotides and proteins. Similarly, Lin et al. [296], reported a plasmonic Doppler grating (PDG) platform for sensing changes in the surrounding refractive index (Figure 10I). The flake‐based Doppler grating has also been demonstrated as a useful platform for quantifying coking layer thickness and for spectrometer‐free optical hydrogen sensing [305, 306]. Further reported applications of gold flakes in sensing platforms are summarized in Table 3.

### 5.4 Scanning Probe Microscopy

Owing to their nanometric thickness, large‐area gold flakes exhibit partial transparency, making their surfaces accessible for both photons and electron beams from both the front and the back side [6, 37, 309]. This makes them ideal substrates for various scanning probe microscopy (SPM) techniques, including scanning tunneling microscopy (STM), where the substrate has to be conductive [309, 310], and tip‐enhanced Raman spectroscopy (TERS), where tip and flake form a plasmonic gap mode [311, 312, 313]. They allow for investigations of adsorbed species down to the single molecule resolution, providing insights into binding sites and molecular orientations, and can additionally benefit from the chemical information using tip‐enhanced Raman spectroscopy (TERS; seminal paper, not using flakes [314]:). Indeed, gold flakes were utilized first as flat substrates for investigating atoms and small clusters adsorbed on the flake surface using electron microscopy [315].

**FIGURE 11:**
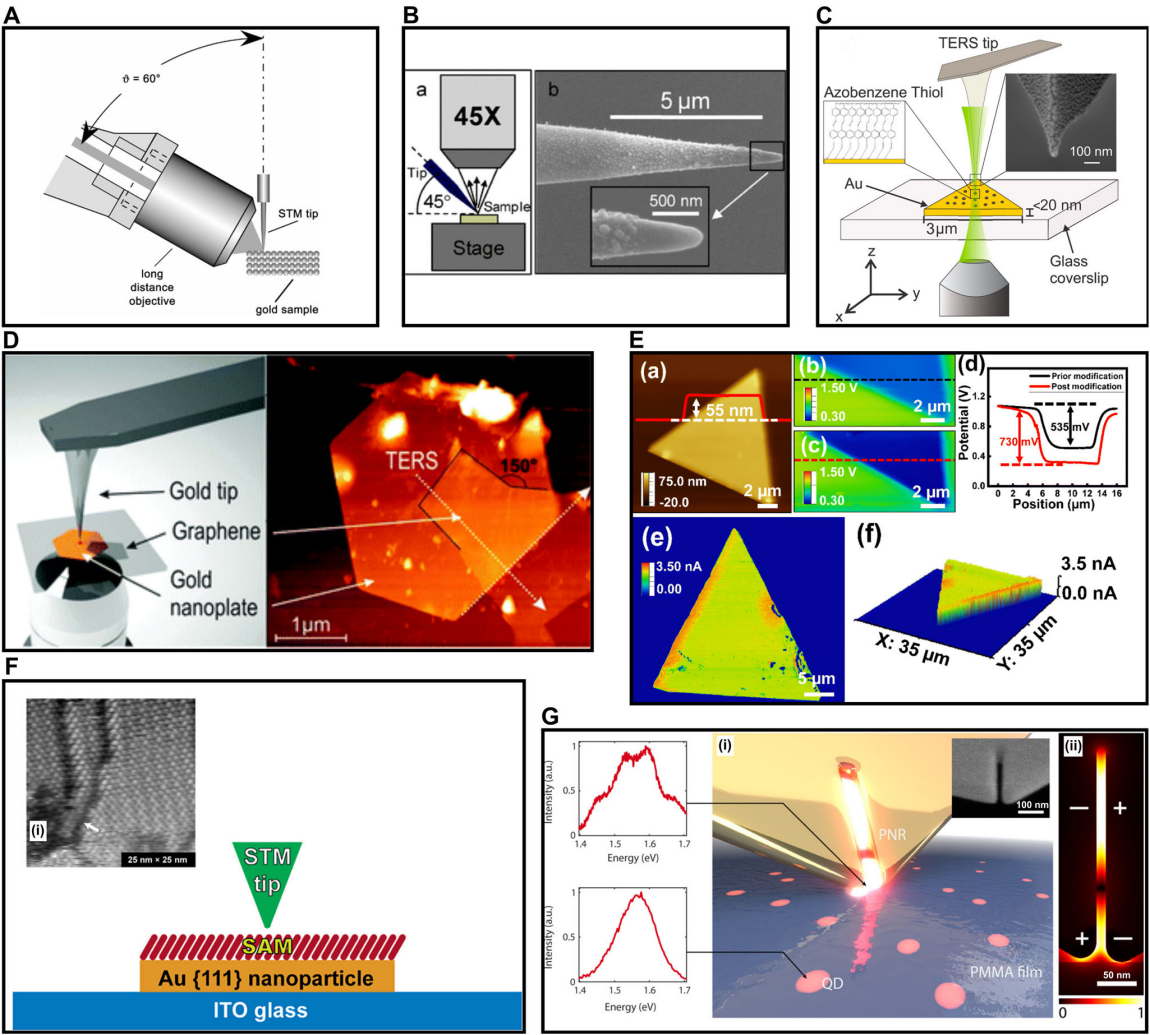
Au flakes in scanning probe microscopy. (A)–(C) Different experimental setup arrangements of the gold flake platform in TERS and STM. (A) Tip setup using a 60° arrangement. Panel A reprinted with permission from Pettinger et al. [316], 2004 American Physical Society. (B_a_) vertical configuration setup. (B_b_) SEM images of the tip. Panel B reproduced from Pienpinijtham et al. [330], with permission from the Royal Society of Chemistry. (C) Setup in back‐reflection geometry, showing the gold flake and self‐assembled monolayer (SAM). The inset displays an SEM image of the tip. Panel C reprinted (adapted with permission from Pashaee et al. [317]. Copyright 2013, American Chemical Society. (D) TERS setup illustrating the gold flake and graphene on the left, with AFM images on the right showing a gold nanoplate partially covered with graphene. Panel D reproduced from Pashaee et al. [318], with permission from the Royal Society of Chemistry. (E) Electrical characterization studies and tunneling current imaging for Au flake. (E_a_) AFM topography of triangular Au flake. (E_b_)‐ (E_d_) Surface potential characterization. (E_e_)‐ (E_f_) HD‐Kelvin probe force microscopy and contact‐AFM characteristics of SAM‐modified Au flake for electrical output performance. Panel E reproduced from Zhang et al. [310], with permission from the Royal Society of Chemistry. (F) Schematic of a Scanning tunneling microscopy (STM) setup and high‐resolution imaging of a gold flake surface functionalized with a SAM. The corrugation is the √3 × √3 R30° molecular lattice characteristic of well‐ordered alkanethiol SAMs, with dark features (arrow) indicating structural domain boundaries. Panel F reprinted (adapted) with permission from Dahanayaka et al. [309]. Copyright 2006, American Chemical Society. (G) Strong coupling via precise nano positioning of a gold flake‐based resonator probe. (G_i_) Illustration of the gold flake‐based probe interacting with QDs (quantum dots) embedded in a polymer film. Left panel: The spectrum of a QD changes significantly when coupled to the slit‐like probe at the tip apex. Inset: SEM image of a nano resonator at the apex of a probe tip. (G_ii_) Map of the electric field distribution of the resonator mode used in the experiment. The + and − signs indicate the instantaneous charge distribution, highlighting the mode's weakly radiative quadrupolar character. Reprinted (adapted) with permission from Groß et al. [212]. Copyright 2018, The American Association for the Advancement of Science.

TERS or STM benefit from the ability to maintain a few‐ to sub‐nanometer separation between the probe tip and the gold flake surface, achieved through feedback mechanisms based on tunneling currents or mechanical forces. Possible experimental configurations are shown in Figure 11A–C. Ren et al. [311], studied thiol molecules adsorbed in self‐assembled (sub)monolayers, while Pettinger et al. [316], (Figure 11A) used TERS to investigate several different analyte species physisorbed on gold flakes. Deckert et al. [312], utilized flat crystalline gold flakes for label‐free investigations of biomolecules using a back‐reflection geometry setup, as depicted in Figure 11C. This approach allowed the simultaneous collection of molecular and topological information. In a related study, Pashaee et al. [317], employed a back‐scattering TERS geometry with a polarized light source to investigate thiolated molecular species, revealing the polarization dependency of the excitation light at the tip/substrate interface. Additionally, Pashaee et al. [318], characterized the edges of graphene‐like and graphitic platelets composed of a few layers of graphene deposited on a gold flake substrate (Figure 11D). The setup also enabled a quantitative investigation of surface plasmon resonance (SPR) and near‐field (NF) heating experienced exclusively by molecules directly contributing to the TERS signal [313, 317, 318].

STM studies have highlighted the unique advantages of gold flakes for high‐resolution surface analysis. Zhang and colleagues [310], realized an improved friction‐driven triboelectric generator by utilizing the atomically flat surface together with a self‐assembled monolayer (SAM) interacting with an STM tip (Figure 11E). Similarly, Dahanayaka et al. [309], demonstrated the use of flake for SAMs of alkanethiols, capturing structural details at the atomic level by high‐resolution STM imaging (Figure 11F_i_). In addition, triangular gold flakes have been used to probe the contribution of plasmonic modes to the local electromagnetic density of states under STM excitation [319]. It is also possible to realize a tip for scanning optical nearfield microscopy (SNOM) from gold flakes, as shown by Groß et al. [212], (Figure 11G). A slit resonator was cut by means of Ga FIB into a gold flake edge after its transfer to an AFM cantilever. This even allowed the establishment of strong coupling to a mesoscopic colloidal quantum dot, opening the possibility for ultrafast coherent manipulation of the quantum dot–plasmon system under ambient conditions [212, 216]. Reported studies integrating gold flakes into SPM platforms are summarized in Table 4.

### 5.5 Catalysis

**FIGURE 12:**
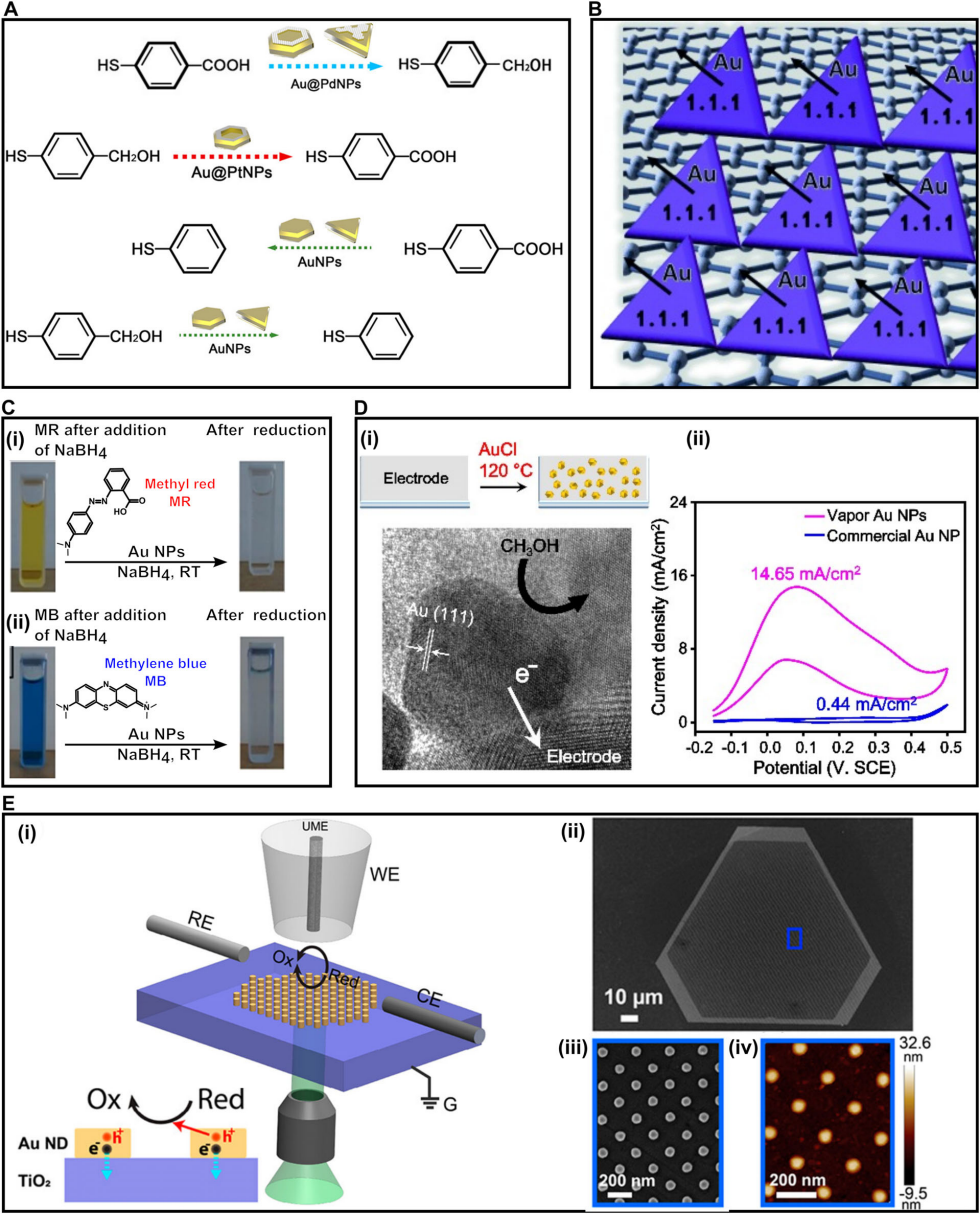
Gold flakes in heterogeneous catalysis. (A)Monometallic and bimetallic gold flakes in different plasmon‐driven oxidation and reduction catalysis. Reprinted with permission from Li et al. [328], Copyright 2021, American Chemical Society. (B) Catalytic platform of oriented gold flake supported on Graphene. Reprinted with permission from [324]. Copyright 2016, WILEY‐VCH Verlag GmbH & Co. KGaA, Weinheim. (C) Catalytic degradation of Methyl Red (MR) in (C_i_) and Methylene Blue (MB) in (C_ii_). Both panels show the change in color before and after the reaction. Panel C reprinted with permission from Bhosale et al. [333]. Copyright 2016, WILEY‐VCH Verlag GmbH & Co. KGaA, Weinheim. (D_i_) Au flake catalytic activity toward methanol (D_ii_) comparison of flakes and commercial particles in the catalysis of methanol. Panels (D) reprinted from Yang et al. [105]. (E) Plasmonic‐assisted catalysis platform. (Ei) Schematic of the designed plasmonic heterostructure and the light‐assisted scanning electrochemical microscopy configuration, with a side‐view schematic illustrating the direction of carrier transfer at the interfaces. (Eii) SEM image of the fully fabricated pattern in Au, with a higher‐magnification view shown in (Eiii). (Eiv) AFM image of the fabricated Au nanodisk array shown in (Eiii). Panel E adapted from Kiani et al. [334]. Copyright 2023, The Authors. Published by the American Chemical Society. This publication is licensed under CC BY 4.0.

Gold nanostructures are well‐known for their heterogeneous catalytic activity [275, 320, 321, 322, 323, 324], a remarkable property that emerges exclusively at the nanoscale [325]. Compared with other nanoparticle morphologies, nanoplates and platelets exhibit exceptionally high surface‐to‐volume ratios, a high density of low‐coordinated atoms along their perimeters, and relatively elevated surface energies, features that are well known to enhance catalytic activity and selectivity across a range of applications [326]. The abundance of coordinatively unsaturated gold atoms at edges, corners, and step sites promotes efficient electron transfer of spontaneously bound molecules, thereby facilitating catalytic turnover [326]. In addition to their high aspect ratio, reducing nanoplate thickness further enhances catalytic performance by increasing the fraction of accessible active sites and amplifying surface‐dominated effects [326]. Together, these factors highlight the complex interplay between thickness, crystallographic orientation, defect density, and local atomic coordination in governing catalytic behavior in gold nanoplates [4]. The catalytic response is also strongly influenced by the surrounding environment and support interactions. For example, gold exhibits particularly high activity when supported on reducible oxides such as TiO_2_, where interfacial charge transfer between the metal and the oxide can occur. Moreover, surface modification can alter site‐specific reactivity: Au nanoplates coated with SiO_2_ have been reported to exhibit enhanced activity at corners and edges, whereas bare Au nanoplates without a SiO_2_ coating show comparatively higher activity on flat surface facets under similar reaction conditions [4]. Coupling platinum with gold to create a gold–platinum bimetallic flake structure increased the catalytic reaction selectivity compared to monometallic gold flakes [327, 328], (Figure 12A). Finally, it is known that plasmonic resonances can enhance the (photo‐)catalytic properties by concentrating photons to reaction sites, enhancing the yield of hot electrons available to drive the reaction, and elevating the reaction site temperature [329]. By precisely controlling the size, shape, and preferential facet orientation of gold nanostructures, their catalytic performance can be significantly enhanced and tailored for specific reactions [322, 323, 324]. For instance, Primo et al. [324], developed a hybrid catalyst by incorporating large‐area gold flakes with a (111) facet orientation into a graphene matrix (Figure 12B. This configuration exhibited superior catalytic activity compared to non‐oriented gold structures, enabling a range of reactions, including selective oxidations, reductions, and coupling reactions.

In a comparative study, Goyal et al. [276], evaluated the catalytic performance of spherical gold nanoparticles and large‐area gold flakes in the oxidation of dopamine and ascorbic acid. The gold flakes demonstrated higher catalytic activity, likely due to the large exposed (111) lattice planes. Similarly, gold flakes were assessed in the degradation of azo compounds such as methylene blue, where they showed exceptional catalytic activity (Figure 12C) [333]. Furthermore, He et al. [293], and Li et al. [279], reported that gold flakes effectively catalyze the decomposition of H_2_O_2_ to O_2_. He et al. also reported high efficiency and selectivity in the catalytic hydrogenation of α,β‐unsaturated aldehydes, and Momeni et al. [278], demonstrated efficient electro‐catalysis of formic acid on carbon ionic liquid electrodes modified with gold flakes.

Wenjing and his group explored the field of catalysis by harnessing the gold flakes as substrate electrode to construct bimetallic and trimetallic Au‐based catalysts [277], with platinum (Pt) or palladium (Pd) deposited on the gold surface. These hybrid catalysts exhibited enhanced catalytic activity for methanol electrooxidation and demonstrated greater resistance to catalyst poisoning compared to commercial Pt‐based catalysts. Similarly, methanol oxidation was obtained by Yang et al. [105], by employing a vapor‐phase synthesis method to deposit the gold flakes onto fluorine‐doped tin oxide (FTO) substrates, achieving significantly higher methanol oxidation rates compared to Au‐deposited nanoparticles on the same FTO platform, as shown in Figure 12D. As they can be transferred to electrodes, electrocatalysis applications are a future research direction to be expected. Structured flake surfaces also hold promise for controlling photochemical reactions. Kiani et al. [334], showed that patterned nanoantenna arrays fabricated within the flake can drive photocatalytic reactions, with plasmon excitation, hot‐carrier generation, transport, and interfacial collection collectively promoting the oxidation of ferrocyanide molecules (Figure 12E). A summary of reported studies integrating gold flakes in catalysis is provided in Table 5.

### 5.6 Other Applications

**FIGURE 13:**
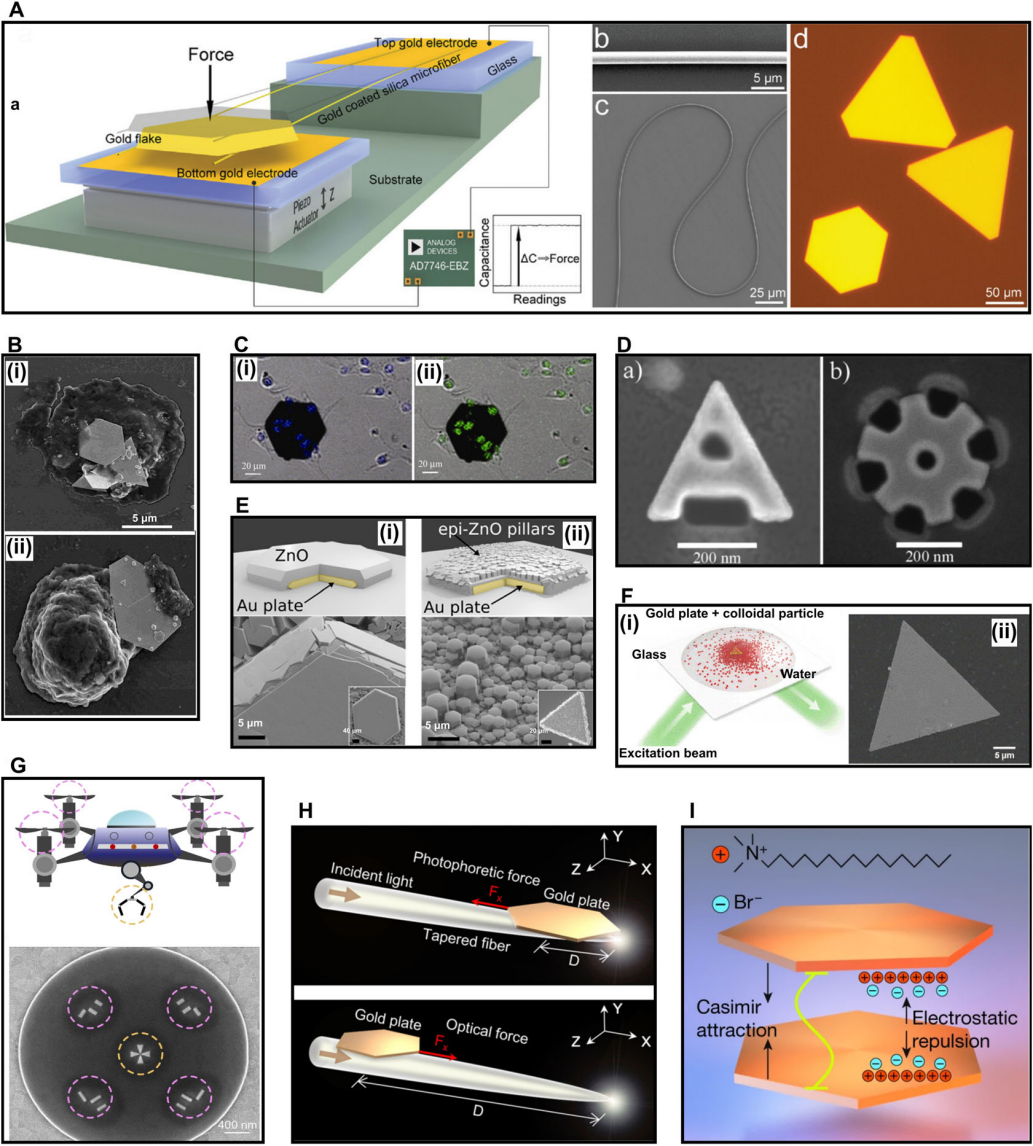
Gold flakes in various fields. (A_a_) Schematic diagram of the miniature capacitive pico‐balance. (A_b_), (A_c_) SEM images of straight and bent silica microfibers. (A_d_) Optical micrograph of single‐crystal gold flakes. Panel A reprinted with permission from Zhu et al. [336]. Copyright 2024, Wiley‐VCH GmbH. (B_i_) SEM image of cells holding a gold flake with a membrane cup. (B_ii_) Partially phagocytosed gold flake. Panel B reprinted (adapted) with permission from Singh et al. [337]. Copyright 2014, American Chemical Society. (C_i_), (C_ii_) Confocal images of a gold flake hosting mouse 3T3 cells, stained with Hoechst and Alexa 488 to visualize the nucleus and the acetylated histone H3, respectively. Panel C adopted from Radha et al. [342]. Copyright 2010, Tsinghua University Press and Springer‐Verlag Berlin Heidelberg. (D) Various nanocomponents, including nanogear, fabricated from gold flakes. Panel D reprinted (adapted) with permission from Ah et al. [344]. Copyright 2005, American Chemical Society. (E_i_) Schematic of flat ZnO on gold flake, showing the Au substrate beneath a smooth ZnO film, and an SEM micrograph of the smooth morphology of microwave‐nucleated ZnO after growth. (E_ii_) Nucleation and growth steps of oven‐nucleated ZnO on gold, with an SEM micrograph showing rough, faceted morphology after the growth. Panel E reprinted (adapted) with permission from Joo et al. [352]. Copyright 2013, American Chemical Society. (F_i_) Schematic illustration of colloidal particle assembly on and around a gold flake. The flake is represented as a yellow triangular plate, and the colloidal particles as red spheres initially dispersed in solution. Arrows indicate the direction of excitation. (F_ii_) Field emission scanning electron microscopy (FESEM) image of a representative gold flake used in the experiments. Panel F was reused with permission from [353]. Copyright 2020, IOP Publishing Ltd. (G) Micro drones with embedded optical tweezers. The upper panel depicts a conceptual schematic of the microrobot design, which integrates individually addressable motors for precise navigation and an independent gripping mechanism. The lower panel shows SEM image of the fabricated microrobot. The dashed circles in the upper and lower panels correspond to structural features marked by the purple and yellow dashed circles in the lower panel. Panel G reprinted from Qin et al. [355]. Copyright 2025, The Author(s). (H) Schematic of pulling a hexagonal gold plate up on a tapered fiber near the tapered fiber end in the upper scheme and pushing the same gold plate back in the middle section of the tapered fiber in ambient air. The tapered fiber is guided with unpolarized supercontinuum light. Panel (H) reprinted with permission from Lu et al. [359], 2017, American Physical Society. (I) A schematic sketch of the system showing two parallel gold nanoflakes floating in an aqueous solution thanks to the joint action of the attractive Casimir force and the repulsive electrostatic force. Panel (I) reprinted from [360]. Copyright 2021, The Author(s), under exclusive license to Springer Nature Limited.

Gold flakes have also found applications beyond the conventional realms of optics, sensing, and electronics. For example, Yu et al. [335], and Zhu et al. [336], realized a miniature capacitive balance with a piconewton‐level detection limit, suitable for measuring microscale object masses or weak forces (Figure 13A). In this design, the gold flake is suspended and serves as a mirror within a laser setup, achieving a detection limit as low as a few hundred nanograms.

Gold flakes have also shown remarkable biocompatibility. Singh et al. [337], investigated the interaction between gold flakes and mice peritoneal macrophages using live‐cell confocal imaging and SEM (Figure 13B). Their study revealed that cells can selectively interact with gold flakes, either by internalizing them or exhibiting a frustrated phagocytosis‐like phenomenon, particularly with larger gold flakes that cannot be internalized. This cellular interaction has been harnessed in drug delivery applications, where biocompatible gold flakes were loaded with drugs showing a sustained release, leading to antitumor activity in the cell line and suggesting their potential as novel drug carriers for breast cancer therapy. The tunable optical properties of large area flakes also enable their use as NIR‐absorbing films or antennas, as reported by Shankar et al. [131], with a possible integration of the material into medical applications, e.g., hyperthermia treatment of cancer cells and in IR‐absorbing optical coatings [52, 131]. Another biological application was reported by Radha et al. [342], who leveraged the biocompatibility and fluorescent signal enhancement properties of gold flakes for single‐cell studies (Figure 13C). By positioning cells in proximity to the gold surface, they demonstrated an enhancement of fluorescence signals from fluorophores within the cells, allowing them to probe the dynamic interactions between cells and their microenvironment.

On a smaller scale, plasmonic optical antennas have successfully enabled single‐molecule biochemical investigations [343]. The antenna serves as an anchoring site for mechanically interlocked molecules. Gold flakes have also been introduced as versatile nanoscale building blocks. Ah et al. [344], reported the fabrication of nanocomponents and nanomachines, such as a nano wheel capable of moving by a few microns, fabricated using top–down strategies such as FIB and EBL (Figure 13D), exemplifying the convergence of “top–down” and “bottom–up” approaches in nanotechnology. Similarly, Yun et al. [345], fabricated nanoscale components from gold flakes – including nano blocks – that could be used in nanoelectromechanical systems (NEMS), paving the way for the development of nano/micro‐machines like nano wheels and nano saws.

In materials science, the physical properties of gold flakes were exploited shortly after the discovery of the flakes, for investigating the epitaxial growth of metals such as iron on the flake surface [48, 315, 346, 347, 348, 349, 350, 351]. Later, Joo et al. [352], utilized the surface area of the flake for the heteroepitaxial deposition of continuous ZnO films through solution‐based techniques (Figure 13E), allowing detailed studies of metal–semiconductor junctions, photovoltaics, and new devices. Meanwhile, Sharma et al. [353], employed gold flakes as antennas for the optothermal assembly of colloidal silica microparticles near the flakes to explore the controlled, two‐dimensional assembly of colloidal crystals through thermal gradients, contributing to the understanding of light‐activated colloidal matter (Figure 13F). An even more exotic application is special optical antennas for efficient photon momentum transfer, used by Wu et al. [354], to create light‐driven microdrones, later enhanced with embedded optical tweezers for nanoparticle manipulation [355], (Figure 13G). Gold flakes serve as substrate for optomechanical systems, enabling rotary wave‐driven motors [356], flake opto–thermo–mechanics [357, 358], and optomechanical light‐induced pulling and pushing through the synergistic interplay of optical and photophoretic forces (Figure 13H) [359]. In a related approach, Munkhab et al. [360], employed gold flakes instead as a near‐ideal reflective surface for investigating Casimir forces (Figure 13I) (recent review about Casimir‐Lifshitz Optical Resonators can be found here: [361]), where the quantum vacuum fluctuations of photons inside a cavity are altered, leading to a very small force on the cavity mirrors. Finally, the thermal properties of gold flakes have also been explored in hybrid nanocomposite materials for tuning the photothermal properties, security printing applications, and even traditional drawing pigments [291, 362], further emphasizing the vast potential of this unique 2D material. Additional examples of gold flake applications beyond the main categories discussed above are summarized in Table 6.

## 6 Monocrystalline vs. Polycrystalline Gold

**FIGURE 14:**
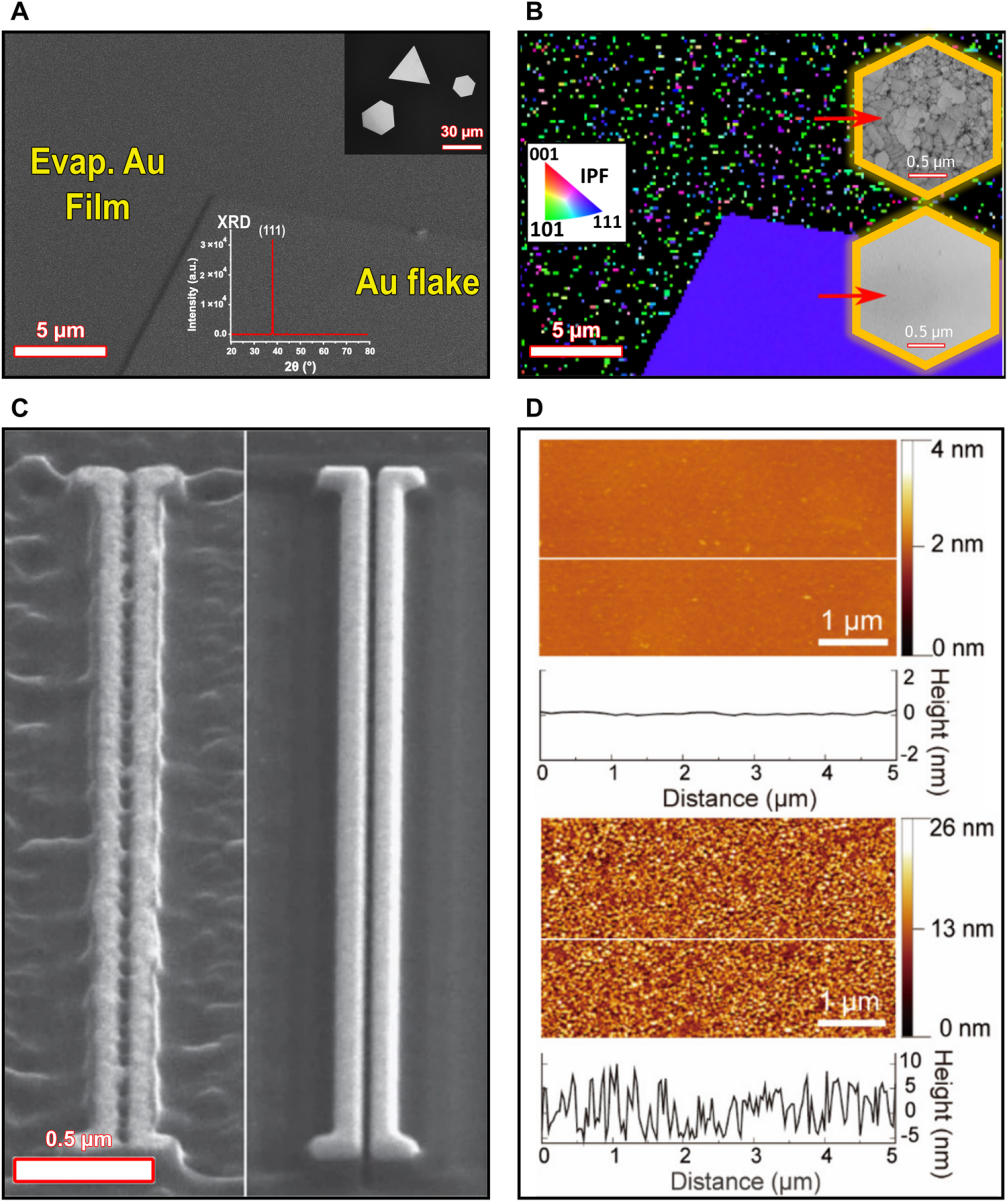
Monocrystalline versus polycrystalline gold structures and structural characterization. (A) SEM images of chemically synthesized monocrystalline Au flakes deposited on an evaporated polycrystalline Au film. The upper inset shows a zoomed‐out view of multiple gold flakes with different shapes. The centered lower inset presents the corresponding XRD diffractogram, dominated by the Au (111) reflection at 2θ = 38.1°, indicating the preferred plate‐like (111) orientation parallel to the supporting substrate. (B) Inverse pole figure (IPF) maps derived from EBSD (electron backscatter diffraction) measurements and superimposed on the corresponding SEM image in (A). The maps confirm that the chemically synthesized flakes are monocrystalline, whereas the underlying evaporated gold film is polycrystalline, exhibiting multiple crystallographic orientations and grain boundaries. The stereographic triangle of the IPF color scale is shown on the left, while a higher‐magnification SEM view of the surface is displayed on the right for each surface. Panels (A), (B)Reprinted from Sweedan et al. [36]. Copyright 2024, The Authors. Small, published by Wiley‐VCH GmbH. (C) Optical nanocircuits fabricated using FIB on a vapor‐deposited multi‐crystalline (left) and a monocrystalline gold flake on the right. Panel (C) reprinted with permission from Huang et al. ref. [6]. Copyright 2010, Springer Nature Limited. (D) AFM images and line scans along the indicated white lines for a deposited gold film and monocrystalline gold flake. Panel (D) Reprinted (adapted) with permission from Liu et al. [183]. Copyright 2022, American Chemical Society.

The investigation of grain‐boundary loss channels in polycrystalline gold is not new [363, 364, 365]. In this context, the atomically smooth grain‐boundary‐free nature of monocrystalline gold flakes (Figure 14A,B) provides distinct and measurable advantages over conventional and easier accessible evaporated polycrystalline gold films [6, 35, 36, 37, 147, 167, 183, 188, 198, 249, 255, 366, 367]. Electron scattering at crystallographic defects and surface roughness is close to the theoretical minimum, leading to large plasmonic wave decay lengths and relatively small plasmonic resonance widths, also close to the theoretically optimal values. In addition, the fabrication fidelity is not spoiled by domain borders and crystallite orientation‐dependent milling/etching resistances, the mechanical properties are homogeneous and reliably optimal, and both optical and electronic performance are enhanced in general [6, 35, 36, 37, 147, 167, 183, 198, 249, 255, 366], (Figure 14C). Quantitatively, Wu et al. [249], reported, e.g., that nano disk arrays fabricated from monocrystalline gold flakes achieve a significantly higher plasmonic quality factor (Q = 23.3) compared to polycrystalline films (Q = 15.1), together with a deeper resonance dip (minimum transmittance 41.3% vs 62.2%). Wiley et al. [198], demonstrated that single‐crystalline gold nanowires show approximately threefold lower radiative losses and support plasmonic resonator behavior not attainable in polycrystalline nanowires. Monocrystalline gold nanoantennas are especially useful in higher‐harmonical optical processes, e.g., exhibiting more than 10^2^ higher two‐photon‐excited photoluminescence intensity under resonant excitation compared to polycrystalline counterparts, indicating substantially stronger local field enhancement [6]. Boroviks et al. [148], observed that meta‐surfaces patterned within monocrystalline flakes display improved efficiency (∼5% enhancement) and enhanced second‐harmonic generation (SHG) by ∼3.5× compared to identical structures fabricated in polycrystalline films, reflecting anisotropic surface properties unique to single crystals [255]. Material quality becomes increasingly important as device thickness decreases. In the ultrathin regime, Pan et al. [37], demonstrated improved electrical continuity and optical performance in monocrystalline gold. At a thickness of 9 nm, the sheet resistance of monocrystalline gold was approximately 9 Ω/sq, compared to 31 Ω/sq for polycrystalline films of identical thickness. Moreover, while polycrystalline films' conductivity deteriorates below ∼7 nm due to percolation limitations, monocrystalline flakes conserve the conductivity down to at least 1.4 nm thickness. Correspondingly, localized surface plasmon resonances (LSPR) are vanishing in polycrystalline films below this threshold, contrary to monocrystalline flakes. Optical transparency further reflects this distinction: at 2.5 nm thickness, monocrystalline gold exhibits ∼91% transmittance near 600 nm, compared to ∼70% for polycrystalline films due to additional scattering and absorption [37]. Similarly, Qin et al. [35], showed that surface plasmon polaritons supported by monocrystalline Au nanosheets exhibit propagation lengths approaching theoretical limits, representing an average of ∼700% increase relative to polycrystalline films. This improvement correlates with substantially lower surface roughness (RMS ≈ 0.25 nm vs 0.9 nm) and no visible domain boundaries. Additionally, Prämassing et al. [238], reported significantly longer propagation lengths for monocrystalline gold in slot structures with widths below 100 nm, while Kuttge et al. [372], reported a scattering coefficient of 0.2% for polycrystalline gold. The surface atomically flat surface was confirmed as well by Liu et al. [183], with RMS ≈ 0.2 nm vs 4.1, 2.7, 1.4, and 1.1 nm for films with thicknesses of 15, 25, 53, and 100 nm, respectively (Figure 14D). Karaman et al. [167], further demonstrated crystallinity‐dependent ultrafast carrier dynamics in 10‐nm‐thick films. While both systems behave similarly at low fluence, monocrystalline films exhibit distinct early‐stage thermalization behavior at higher excitation levels. The extracted electron–phonon coupling constant was G = (2.2 ± 0.1) × 10^1^ ^6^ W·m^−^^3^·K^−^^1^ for monocrystalline gold, compared to G = (2.0 ± 0.1) × 10^1^ ^6^ W·m^−^^3^·K^−^^1^ for polycrystalline films. In addition, hot‐electron injection into TiO_2_ reached ∼9%, approaching theoretical limits (∼10%), highlighting efficient interfacial carrier extraction in the grain‐boundary‐free platform. Additional plasmonic studies support these trends. Liu et al. [183], reported that nanoparticle‐on‐mirror nanocavities fabricated from monocrystalline gold flakes exhibit approximately twofold higher quality factors and threefold stronger scattering intensity than polycrystalline analogues. In the same context of using the flake as a mirror, the flake produced a 143‐fold fluorescence enhancement and a 5.4‐fold reduction in the lifetime of crystal violet molecules, which are approximately 6 and 3 times greater, respectively, than the corresponding values obtained on a polycrystalline gold film [188]. From a mechanical perspective, Yi et al. [366], revealed that polycrystalline‐based nanostructures exhibit intrinsic acoustic damping with a quality factor of Q ≈ 11.3 ± 2.5, indicative of grain‐boundary‐dominated dissipation, whereas monocrystalline systems show reduced intrinsic damping contributions and a quality factor of 22.0 ± 2.4. In SERS applications, Sweedan et al., demonstrated that monocrystallinegrating structures yield enhancement factors exceeding those of polycrystallineequivalents by more than two orders of magnitude under identical geometries [36], further emphasizing the role of crystallinity inmaximizing electromagnetic field localization and reproducibility. Finally, in optoelectronics, monocrystalline gold structured devices exhibit an order of magnitude higher open‐circuit photovoltage compared to polycrystalline counterparts, among the highest reported photovoltage performances in terms of on‐chip device density and responsivity per unit area [283]. All these findings substantiate the measurable and application‐relevant advantages of monocrystalline gold flakes over conventional polycrystalline films across optical, electronic, and optomechanical domains^1^.

## 7 Metal Flakes Beyond Gold

Early synthetic studies of metal flakes were largely limited to gold and silver; however, nowadays, the advances in crystal growth and colloidal synthesis have enabled a broader range of metals to be realized in flake‐like morphologies [4, 373]. In addition to the coinage metal triad (Au, Ag, Cu), a variety of other centered cubic (fcc) metals, followed by hexagonal close‐packed (hcp) and body‐centered cubic (bcc) crystal families, have been grown in flake shape, comprising approximately 29, 28, and 23 elements. In contrast, metals with lower crystallographic symmetry have been discussed only rarely in the context of metallic flake formation, as has the emerging family of metallene structures [4, 374]. Moreover, several metals remain largely unexplored in this context, including systems such as ytterbium and zinc. Among plasmonic and optical materials, gold, silver, copper, and aluminum are most frequently employed [375, 376, 377, 378, 379, 380, 381].

The selection of a specific metallic flake for device fabrication or functional integration is typically governed by a balance between physical performance, chemical stability, durability, cost, and biocompatibility [378, 382]. For the sake of a fair comparison, the present discussion is limited to metals that have been experimentally demonstrated to yield atomically flat, large‐area platelet structures. In general, gold has emerged as the default material choice due to its chemical stability under ambient conditions, biocompatibility, and the extensive body of literature providing well‐established synthetic routes and further treatment recipes. In contrast, many alternative metals have been explored to a much lesser extent, particularly with respect to the controlled synthesis of large‐area, atomically flat flakes. Nevertheless, in applications where cost reduction becomes a priority, alternative materials may be considered, especially copper for electronic and electrode applications [379, 381, 383]. For many non‐noble metals, however, surface passivation or encapsulation with protective layers is needed to ensure stability and performance under ambient conditions [377, 382, 384, 385, 386]. Aluminum is the most abundant metal on earth and therefore holds considerable promise for low‐cost plasmonic devices, particularly due to its favorable optical response in the blue and ultraviolet region. However, aluminum is prone to rapid oxidation and requires surface passivation shortly after its synthesis to ensure stability. In addition, the controlled synthesis of large‐area aluminum flakes remains challenging, with only a limited number of reports currently including a single demonstration of thin aluminum sheets available to date [387, 388]. Copper is the second most abundant metal with high oxidation tendency and lower plasmonic efficiency in the visible range, although recent methodologies have demonstrated the formation of large‐area, flat copper flakes [381]. However, in terms of oxidation resistance, chemical stability, and biocompatibility, silver or copper are limited [378, 382, 389, 390]. In contrast, in terms of plasmonic usability, copper nanostructures generally rank lowest, being outperformed by gold and, most notably, silver, which exhibits the best plasmonic properties in the visible spectral range [378]. Other metals, such as palladium and platinum, display even lower plasmonic quality factors.

From a cost perspective, aluminum and copper are the most cost‐effective, followed by silver and gold. Although gold entails a higher material cost—which is, anyhow, for nano materials often not the limiting factor—its favorable attributes frequently outweigh the initial expense in practical implementations [36]. Beyond optics and plasmonics, metallic flakes composed of copper, palladium, platinum, rhodium, ruthenium, and iridium have been investigated primarily for catalytic applications, where surface reactivity rather than chemical inertness is desirable [4, 391, 392, 393, 394, 395, 396, 397, 398, 399]. In such contexts, the ultrasmooth inert surfaces characteristic of gold flakes may be less favorable, while more chemically active metals can deliver higher catalytic performance. Palladium flakes, for instance, have been reported in a variety of catalytic processes [391, 392, 396], and platinum‐based platelet structures have also been explored for catalytic applications. However, the synthesis of platinum nanoplates remains challenging, with epitaxial growth on preformed platelets and two‐dimensional template‐confined growth representing the main accessible routes, often yielding structures with limited surface area [373, 400]. Nickel flakes have likewise been demonstrated to show catalytic activity, although their synthesis is complicated by difficulties in achieving complete reduction and by the formation of surface oxide species that must be carefully addressed [401, 402]. Rhodium, one of the rarest and most expensive precious metals, typically exceeding gold in cost, exhibits excellent catalytic activity but poses significant synthetic challenges due to its exceptionally high surface free energy, approximately three times that of gold [4, 393, 394]. Iridium has also emerged as a promising catalytic material, displaying enzyme‐like activity in selected reactions [4]. In addition, large‐area ruthenium flakes have been reported as highly reactive and effective catalysts for a range of heterogeneous and electrocatalytic reactions [395]. In the context of magnetic applications, metallic flakes composed of ferromagnetic materials such as cobalt and nickel exhibit pronounced magnetic anisotropy. This is critical for functionalities including magnetic storage and memory devices [403], where gold lacks intrinsic magnetic ordering and therefore cannot support magnetically active device architectures [401, 402, 404]. Within the family of hexagonal close‐packed (hcp) metals, large‐area metallic flakes have also been demonstrated. Magnesium flakes, for example, have recently emerged as a promising alternative to aluminum for plasmonic applications, as they support tunable plasmonic responses extending from the visible into the ultraviolet spectral region [405, 406]. However, like other highly reactive metals, magnesium suffers from rapid oxidation and chemical instability when compared to widely used plasmonic materials such as gold. These limitations raise concerns regarding stability, reversibility, and long‐term durability, thereby necessitating effective surface passivation strategies. Although surface chemistry and protective coatings may mitigate these issues, moisture absorption and performance degradation remain significant challenges [4]. In contrast, many bcc metals exhibit limited ability to form high‐quality two‐dimensional platelet structures or are intrinsically highly reactive, as exemplified by alkali metals such as sodium and lithium. Metals with other crystal structures, including bismuth and indium, have likewise been only sparsely explored in the context of large‐area metallic flakes, despite reports demonstrating the feasibility of forming platelet‐like morphologies [4]. As such, these material systems remain comparatively underexplored and fall outside the scope of the present discussion, which is restricted to metals reported to yield large‐area flakes.

## 8 Challenges and Perspectives

**FIGURE 15:**
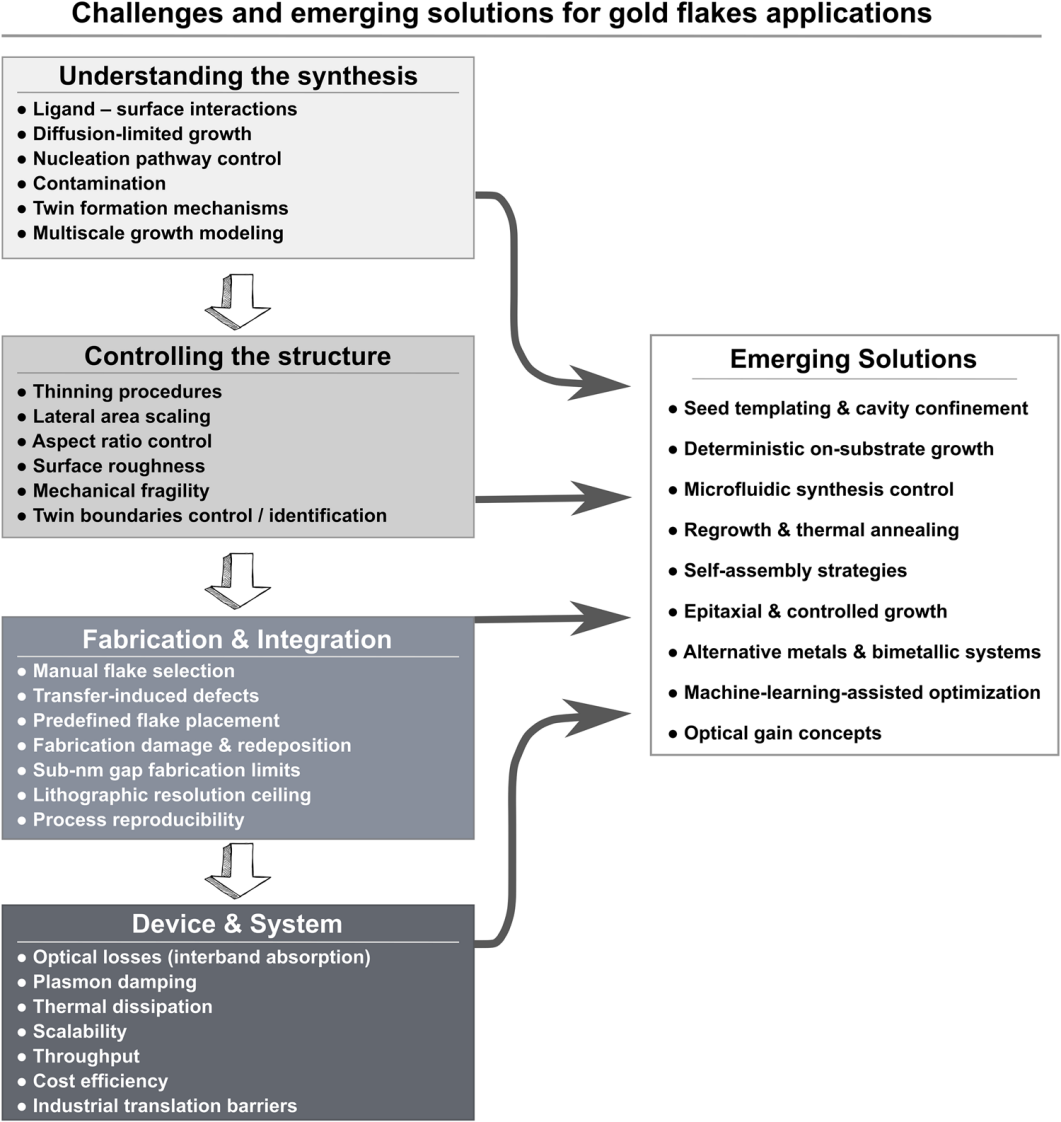
Gold flakes: challenges and future developments. A multiscale overview of the key scientific and technological bottlenecks limiting the implementation of monocrystalline gold flakes, ranging from atomic‐level growth processes to device‐level integration challenges. Arrows indicate hierarchical interdependence between levels and future possible solutions.

Large‐area monocrystalline gold flakes are not yet established in industrial processes, despite the broad range of possible applications demonstrated on the laboratory level. Recent years have seen progress in controlled synthesis and deterministic patterning, but several fundamental and technological challenges remain before their full potential can be realized. Compared to conventional evaporated polycrystalline films, which are sufficient and established for many applications, monocrystalline flakes require more elaborate synthesis and handling. Thus, despite their superior performance, practical implementation demands careful balancing of functional benefits, fabrication complexity, throughput, and scalability. To become fully commercially feasible, the challenges illustrated in Figure 15 must be addressed. They span multiple length scales, from atomic‐level growth mechanisms to device‐level integration constraints, excluding industrial logistics such as large‐scale production infrastructure and quality‐control systems.

### 8.1 Synthesis: Mechanistic Understanding, and Predictive Growth

At the most fundamental level, a deeper understanding of flake growth is still elusive. Although synthesis can be readily performed in standard chemical laboratories, this typically holds only when strict control over lateral dimensions and aspect ratio is not required [6, 10, 36, 54]. Under such conditions, the result is usually a spatially dispersed ensemble of flakes with broad distributions in area and thickness, often accompanied by spherical or irregular byproducts [35, 76]. Accordingly, while laboratory‐level accessibility has clearly been established, translating this accessibility into cost‐effective, uniform, reproducible wafer‐scale production with high throughput remains a major unresolved challenge.

Several mechanistic aspects of flake growth remain insufficiently understood, including surface‐ligand interactions, diffusion‐limited growth, the atomic‐scale origin of twin planes, and effects of surface contamination. Many wet‐chemical syntheses rely on surfactants or polymers such as PVP, halides, or organic capping agents [6, 198], which can remain adsorbed on the gold surface after synthesis. These residual species influence plasmonic properties as well as catalytic activity and other interfacial charge‐transfer processes [407, 408]. Although post‐synthesis cleaning procedures or plasma treatments can partially remove these residues, they may also introduce variability, surface roughening, or secondary contamination.

Critical evaluation of the literature also reveals a limited cross‐comparison between different synthetic strategies. Mechanistic aspects such as nucleation pathways, growth kinetics, ligand–surface interactions, and environmental parameters are typically examined within individual studies, but are rarely analyzed within a unified framework. Consequently, although many approaches achieve excellent structural quality and tunable geometries, overarching design principles governing flake growth remain insufficiently identified. This would enable more rational synthesis design, improved reproducibility, and an enlarged accessible parameter space for gold flakes and also related metallic platelet systems.

Recent advances in atomic‐resolution characterization, particularly cryogenic electron microscopy and in situ techniques, allow direct observation of growth processes with near‐atomic precision. These methods provide an unprecedented opportunity to derive experimentally grounded growth models and to reconcile previously disconnected mechanistic interpretations. However, predictive modeling frameworks capable of reliably forecasting synthetic outcomes remain absent. Established theoretical approaches, including density functional theory (DFT) and molecular dynamics (MD), provide valuable insights into surface energetics and ligand organization on specific crystallographic facets and for very small particles. Their applicability, however, is restricted to relatively small system sizes and limited parameter spaces, rendering full particle–ligand simulations at realistic flake dimensions impractical. A predictive framework would need to integrate thermodynamic and kinetic factors, crystallographic evolution, dimensional growth dynamics, and mass transport in solution, an inherently multiscale problem that remains unresolved.

In this context, data‐driven approaches, particularly machine learning (ML), are promising. The systematic curation of experimental datasets into accessible and standardized databases could enable the training of predictive models capable of correlating synthesis parameters with structural outcomes. Such strategies have the potential to significantly reduce empirical optimization efforts and to accelerate the rational design of gold nanoflake geometries.

### 8.2 Structure: Means for Aspect Ratio Control

Gold flake lateral scaling remains fundamentally limited by the aspect‐ratio. Protocols that increase flake area typically also lead to increased thickness. Consequently, dimensional tunability is governed by growth kinetics and thermodynamic constraints, restricting precise control over flake thickness, lateral dimensions, and spatial positioning relative to lithographically defined architectures. Accepting thicker flakes would compromise one of the main advantages of solution‐grown systems, as additional thinning steps would then become necessary. Current thinning techniques, including etching or polishing, introduce surface roughness, degrading the atomic‐scale homogeneity of as‐grown flakes. Continued refinement of chemical growth strategies is therefore expected to provide a scalable pathway toward large‐area, ultrathin monocrystalline platforms. As an intermediate approach, albeit at the cost of additional steps and processing time, wet‐chemical regrowth or thermal annealing following thinning procedures may partially restore surface flatness and recover access to the quantum and plasmonic properties of ultrathin flakes for research‐scale applications [37, 249, 409, 410].

Several promising directions have emerged to overcome these limitations. One strategy involves restricting nucleation and growth sites through more controlled synthetic approaches, as discussed earlier in this review. The patterned placement of seed crystals in predefined arrays could fix flake position and orientation while narrowing size distributions, whereas defined cavities acting as growth templates may confine lateral dimensions within tightly controlled margins. For example, Capitaine et al. [411], demonstrated a hybrid approach combining bottom–up and top–down strategies, where assemblies of single‐crystal gold nanocubes were epitaxially transformed into continuous monocrystalline plasmonic structures with arbitrary geometries. In addition, epitaxial electrochemical deposition has enabled the growth of atomically flat gold films with tunable thicknesses on prefabricated monocrystalline silver surfaces [412, 413], Magnesium oxide substrate [414], on MoS_2_ surface [415], and of epitaxial hexagonal close‐packed (hcp) gold sheets on graphene [416, 417]. Techniques adapted from the classical silicon–germanium industry, such as the Czochralski process, have been explored for producing thin layers of monocrystalline gold. Vesseur et al. [154], demonstrated that this approach can provide suitable starting materials for photonic applications. Further refinement of conventional methods, such as chemical vapor deposition (CVD) could also advance the field [418]. Likewise, improving template‐stripping techniques, which have been reported to enable the production of high‐quality metal films for plasmonic applications [419, 420], may represent another promising route, even when applied to polycrystalline films.

Deterministic patterning strategies, such as seed‐mediated growth on substrates pre‐patterned via lithographic or vapor‐phase processes, have allowed for the controlled formation of gold flake arrays with defined faceting and orientation [106, 421, 422, 423, 424]. Nanoimprint lithography combined with plasmon‐mediated growth has also shown promise for generating periodic arrays with high precision [421, 422, 424]. Another route toward achieving higher substrate coverage is the recrystallization of evaporated polycrystalline film [249]. Although these methodologies remain in early stages of development, they hold considerable promises for enabling automated device architectures based on monocrystalline gold flakes.

Self‐assembly remains largely unexplored. Flakes synthesized in a charged state, or functionalized to induce controlled interparticle repulsion or attraction, could organize from the bottom up into ordered arrays with tunable spacing, enabling collective optical responses and tailorable plasmonic functionalities [425, 426, 427, 428]. Similarly, gold flakes functionalized with DNA strands are a possible route to enable programmable pattern formation [428], directed assembly into predefined geometries [426], or the formation of macroscopic hierarchical architectures from nanoscale building blocks [150, 286, 425, 427], while simultaneously enriching surface functionality [150, 286].

Post‐synthetic modification can further expand gold flake functionality. Flakes can be structurally or compositionally modified, using the same or a different metal or even hybrid compositions. Future directions include the development of nano‐island hybrid structures, the realization of compositionally and architecturally complex morphologies such as nano frames and hollow flakes, and epitaxial growth of secondary metals to form flakes heterostructures [4, 373]. Continuous‐flow reactors, particularly microfluidic systems with in‐line monitoring, could offer real‐time control over growth dynamics, potentially enabling scalable and reproducible production.

Finally, there are efforts to alter the flakes themselves, as presented by Zhang et al. [32], where during chemical thinning a structural phase transition from fcc to hcp has been observed at thicknesses around 12 nm. This leads to increased mechanical stability and promises even smaller devices to be developed in the future. Likewise, depending on the application, the amount and distribution of twin boundaries may also critically influence performance as they define the minimal achievable flake thickness and shape. At present, however, rapid and non‐destructive quantification of such crystallographic defects within individual flakes remains challenging. Advanced transmission electron microscopy (TEM) techniques, including electron holography, offer potential solutions but are currently expensive and incompatible with high‐throughput screening.

### 8.3 Fabrication and Integration: Handling Improvements

Even when flakes with suitable structural properties are available, fabrication, handling, and integration remain major bottlenecks. In principle, atomically flat monocrystalline gold layers with controlled thickness and lateral dimensions should ideally be generated directly on predefined substrates or even at their final destination as simply as evaporated polycrystalline films. In practice, suitable flakes must often be manually identified and transferred, a process that is slow and labor‐intensive [76, 139]. Microscopy with transmission spectroscopy and motorized stages can partially streamline identification, but full deterministic placement is not yet achieved. Yet, with the advent of automized transfer stage setups, this is within reach.

Subsequent device fabrication steps introduce further constraints. Mechanical delamination, substrate transfer, and nanofabrication procedures can induce cracks, wrinkles, strain, or contamination, particularly in ultrathin flakes [10, 35, 36, 37]. In the few‐nanometer thickness regime, mechanical fragility becomes increasingly pronounced, making the structures highly susceptible to handling‐induced defects. Furthermore, although monocrystallinity eliminates grain boundaries, additional loss channels remain, including FIB‐induced damage, material redeposition during nanostructuring, and processing‐related surface roughness [36, 198, 249].

One particularly promising application of monocrystalline gold flakes is the realization of sub‐nanometer plasmonic gaps, which enable the exploration of emerging quantum plasmonic phenomena. However, the reliable fabrication of such ultrasmall gaps remains technologically challenging. Particle‐on‐mirror geometries currently offer the highest reproducibility, but they inherently limit particle geometries and provide little control over particle positioning. Here, pre‐patterning or seeding schemes are expected to provide viable solutions.

Structuring by means of FIB milling is unlikely to become commercially viable due to its limited throughput. Only the development of multibeam milling systems based on helium ions could potentially improve fabrication efficiency, although this appears unlikely given the declining availability of helium ion microscopes due to a lack of economic incentive. Electron‐beam lithography likewise is unable to fabricate structures with sub‐nanometer resolution in a commercially viable manner. A further step toward improving structural smoothness using existing fabrication routes was reported by Greenwood et al. [429], who demonstrated an EBL‐based reactive ion etching approach that selectively etched gold flakes along specific crystallographic facets, thereby producing smoother sidewalls. The patterning was achieved in a single run within the e‐beam lithography system. Nevertheless, further investigation remains necessary. In the future, additional processing strategies may provide alternative pathways. For example, transferring nanostructures onto a pre‐stretched substrate could reduce interparticle distances in a controlled way while the mechanical tension is released. Nanoimprint lithography represents another potential approach that may reach feature sizes in the few‐nanometer regime when applied to gold flakes; however, current implementations remain limited to approximately 5 nm and require further development. A state‐of‐the‐art overview of these methods is provided in refs. [430, 431, 432], which offers further insights into overcoming remaining barriers in throughput and rapid prototyping.

At present, regrowth strategies suffer from thermodynamic limitations and diffusion inhomogeneities, which could potentially be mitigated by light‐activated growth processes that exploit electromagnetic field concentration within nanoscale gaps. Finally, gaps created by electromigration are not reliably controlled in position or shape, even though initial studies using pre‐patterned gold films have attempted to address these limitations [433].

### 8.4 Device and System: Optics, Catalysis, and Much More

The optical properties of gold show strong absorption for wavelengths smaller than approximately 550 nm, as the photon energy becomes sufficient to excite interband transitions of d‐electrons with an energy of 2.3–2.5 eV. This results in the increased conversion of green and blue light into heat as an additional loss channel, thereby reducing the lifetime of plasmon excitations. This is detrimental for most meaningful applications (although in some cases, heat generation may be beneficial, gold flakes are typically not the material of choice in such scenarios) [434, 435].

There are two primary strategies to address this limitation, each associated with inherent drawbacks. (i) Material substitution: silver or aluminum are metals with low‐loss plasmonic behavior down to the blue parts of the visible spectrum. They are also possible to grow as flakes; however, their areas are not as large as for gold. In addition, they lack stability in ambient conditions compared to gold flakes and must be passivated to ensure long‐term functionality stability (see also our discussion above addressing different metal flakes). (ii) Optical gain; especially for photonics/plasmonics applications, the idea of a repeater, or an optically pumped material which increases the plasmon energy by stimulated emission (spasing, inspired by lasing) is appealing. However, the practical problems of these ideas are demanding, have not yet been solved, and are a topic of actual research.

Once synthesis and fabrication bottlenecks are addressed, monocrystalline gold flakes are expected to play an increasingly important role in catalysis research [436], as well. Catalytic processes in plasmonic systems often involve multiple intertwined physical and chemical mechanisms, including hot‐carrier generation and purely thermal activation pathways, making precise control of experimental conditions essential for mechanistic discrimination [334, 341]. In this context, the geometrical reliability and structural uniformity of monocrystalline flakes may provide a powerful platform for designing tailored architectures that selectively emphasize specific reaction pathways [436]. Beyond gold, alternative metallic flakes (see previous section on non‐gold flakes), as well as bimetallic or hybrid catalytic structures, whether incorporating gold or not, may further expand the accessible parameter space and enable synergistic effects in structure–function performance.

## 9 Concluding Perspective

Overall, the continued development of monocrystalline gold flake platforms will require coordinated progress across multiple length scales, from improved understanding of nucleation and growth at the atomic level to scalable fabrication and deterministic device integration. The major barriers remain tightly interconnected: incomplete mechanistic understanding limits predictive synthesis; constrained predictive synthesis restricts dimensional control, defect engineering, and precise positioning; and insufficient structural control propagates directly into fabrication, handling, and integration challenges. These technological limitations are further compounded by intrinsic material constraints, particularly the optical losses of gold across portions of the visible spectrum. At the same time, the field now has a clearer trajectory than before.

Emerging strategies, including controlled nucleation approaches, hybrid bottom–up/top–down fabrication schemes, microfluidic synthesis with in‐line monitoring, post‐synthetic heterostructuring, self‐assembly, and data‐driven optimization frameworks, are expected to accelerate progress substantially. As these approaches mature, they may enable the reliable production of structurally defined, atomically flat gold nanostructures with tunable geometries and predictable functionality, ultimately establishing monocrystalline gold flakes as a robust platform for next‐generation photonic, electronic, sensing, quantum, and catalytic technologies.

## Conflicts of Interest

The authors declare no conflicts of interest.

## Data Availability

The authors have nothing to report.

## Endnote

^1^There is one work in literature, that shows no measurable difference between polycrystalline and gold and flakes: Kejík, L., et al., *Structural and optical properties of monocrystalline and polycrystalline gold plasmonic nanorods*. Optics Express, 2020 **28**(23): p 34960‐34972. However, the final structures give the impression of too much thermal input during the milling process, leading to very similar kind of annealed geometries. So fabrication methodology matters.
